# Senescent tumor cell membrane–based “time engine” nanosystem amplifies endoplasmic reticulum targeting for metastasis blockade

**DOI:** 10.1126/sciadv.aeh0494

**Published:** 2026-10-09

**Authors:** Jing Tao, Yuzhen Guo, Fengzhi Zhang, Jiahui Wu, Liyun Xing, Jinxia Kong, Lian Li, Zhou Zhou, Yuan Huang

**Affiliations:** Key Laboratory of Drug-Targeting and Drug Delivery System of the Education Ministry and Sichuan Province, Sichuan Engineering Laboratory for Plant-Sourced Drug, West China School of Pharmacy, Sichuan University, Chengdu 610041, China.

## Abstract

As an important component of biomimetic drug delivery systems, cell membrane–coated nanoparticles are widely used for subcellular delivery. However, the influence of cell membranes at different life cycles on subcellular delivery remains unexplored. Our previous study demonstrated that senescent tumor cell membrane–coated nanoparticles (SNP) displayed superior endoplasmic reticulum (ER) targeting efficiency in tumor cells. After inducing tumor cells into senescence, SNP showed greater accumulation in ER. Herein, we design a “time engine” nanosystem for amplified ER targeting: Firstly, ribociclib is encapsulated into SNP (R@SNP) to induce tumor cell senescence; then, SNP loaded with brefeldin A (B@SNP) is used to trigger ER stress. By sequential therapy, R@SNP disrupts the cytoskeleton of 4T1 tumor cells to eliminate ER-targeting barriers and remodels the tumor immune microenvironment; B@SNP boosts ER-targeting efficiency and triggers intense ER stress. Collectively, such time engine nanosystem achieves excellent ER accumulation and antimetastatic efficacy in 4T1 tumor–bearing mice, providing an innovative perspective for subcellular delivery and tumor treatment.

## INTRODUCTION

As an advanced delivery strategy characterized by combined natural attributes and artificial controllability, biomimetic drug delivery systems have garnered widespread research interest in tumor therapy (*1*, *2*). These systems integrate natural transport-mimicking biocomponents (e.g., cell membranes and extracellular vesicles) with programmable carriers (e.g., polymeric nanoparticles, micelles, and inorganic nanocarriers) to achieve prominent synergy (*3*, *4*). Biological components endow the systems with excellent biocompatibility, immune evasion, and lesion-homing properties; nanocarriers, by contrast, offer precise structural designability and controllable drug release profiles (*5*–*7*). Their integration not only prolongs in vivo drug circulation time markedly but also boosts targeted delivery efficiency (*8*–*10*).

Cell-specific physiological remodeling occurs in cells at different life cycles (*11*–*14*). This life cycle–based dynamic transition profoundly affects cells’ migration trajectories, functional activity, and ultimate fate in vivo, which can be exploited for the design of biomimetic drug delivery systems (*15*, *16*). For instance, immune cells and blood cells undergo distinct physiological behavior remodeling during senescence. Senescent neutrophils exhibit marked up-regulation of the C-X-C chemokine receptor 4 (CXCR4) on their cell surface, enhancing their tropism toward bone marrow tissue (*17*). Senescent red blood cells, by contrast, undergo alterations in membrane architecture and surface antigenic markers, become specifically recognized by macrophages, and exhibit splenic homing properties (*18*). These demonstrate that leveraging the distinct physiological behavior of such cells may realize the drug delivery to target tissues. Although several studies have used live intact cells as “hitchhiking” carriers, the cellular transport and underlying mechanisms of such cell carriers, the alterations in drug activity during delivery, and the impact of intact cells on the organism remain to be fully elucidated.

In previous work, our research group constructed a homologous tumor cell membrane–coated nanodrug delivery system based on the “biomimetic cloak” strategy. We first found that this system not only exerted a homing effect on homologous tumors and high affinity for tumor cells but also achieved specific accumulation in the endoplasmic reticulum (ER)–Golgi apparatus membrane system during intracellular trafficking. Moreover, we clarified that its mechanism relied on caveolin-mediated endocytosis and SNARE protein–mediated membrane fusion of homologous cell membranes (*19*–*21*). This raises an important question: What is the subcellular drug delivery capability of cell membranes from cells at different life cycles? Against this backdrop, we further explored the potential of senescent tumor cell membranes for ER-targeted delivery. We found that senescent tumor cell membranes exhibited markedly stronger ER-targeting and retention abilities than normal ones, and senescence induction can reinforce this effect via cytoskeleton disruption. To the best of our knowledge, these findings have not been previously reported. Therefore, leveraging cell membrane biomimetic modification technology and targeting the regulatory characteristics of the “senescent phenotype-ER delivery efficiency-immune response” across distinct tumor cell life cycles, we constructed a time engine nanosystem with both ER-targeting and vaccine-mimicking properties (Fig. 1). This system precisely delivered ER stress inducers to the ER of senescent 4T1 cells to trigger robust ER stress and immunogenic cell death (ICD); it also enhanced antigen presentation by supplying exogenous major histocompatibility complex class I (MHC-I) molecules; promoted the secretion of interferon-γ (IFN-γ), interleukin-1β (IL-1β), and tumor necrosis factor–α (TNF-α); and efficiently activated dendritic cell maturation and T cell immune responses. In vivo experiments verified that this strategy potently suppressed the growth of orthotopic breast cancer in mice, with nearly undetectable lung metastases, and that senescent tumor cell membranes exhibited no overt toxicity to normal liver and spleen tissues. Overall, this study reveals that cell membranes at distinct life cycles have markedly divergent delivery capacities, thereby providing prospective insights and strategies for the rational design of biomimetic drug delivery systems.

**Fig. 1.**
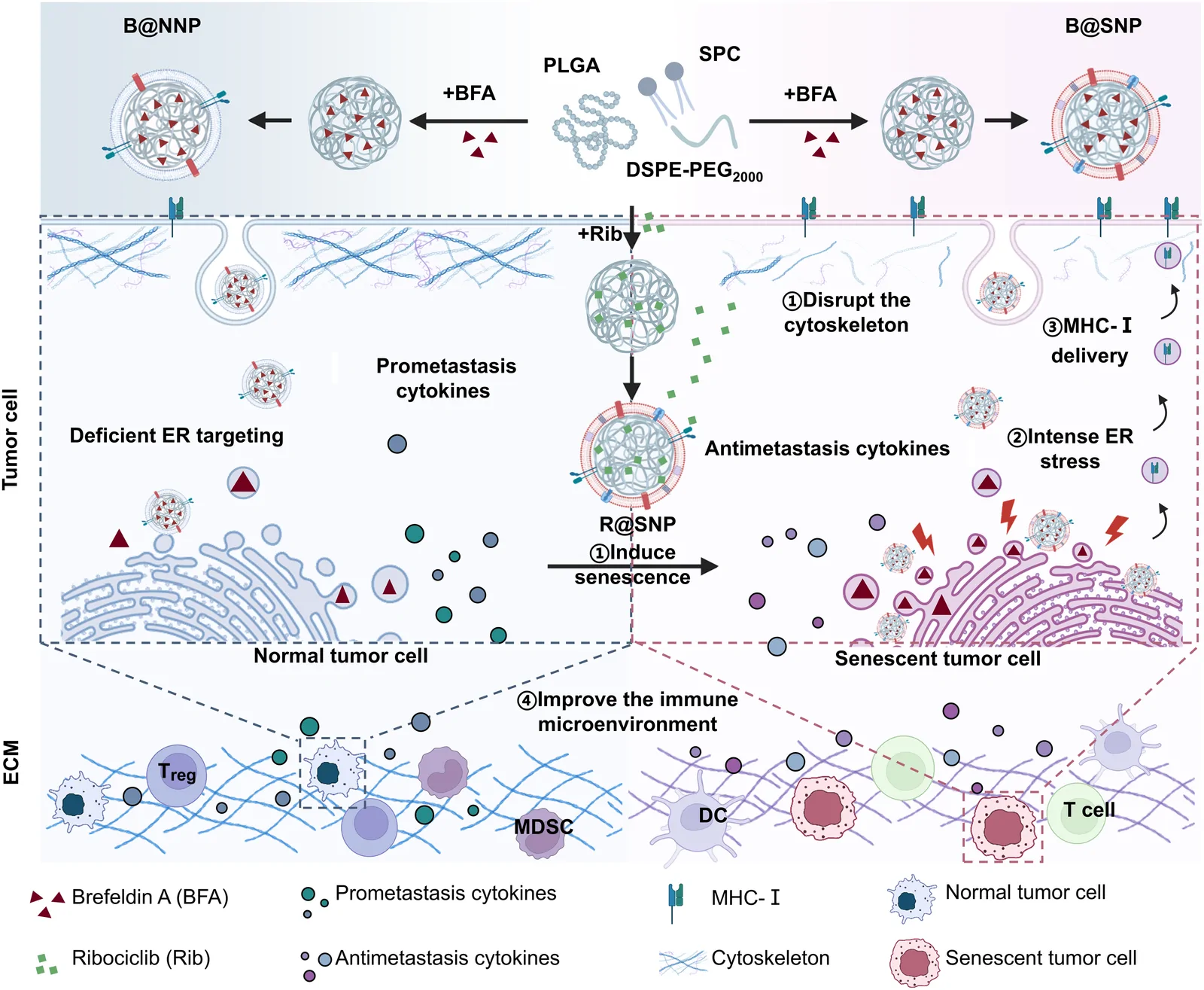
Schematic diagram of the time engine nanosystem. ① Senescence inducer R@SNP induces tumor cell senescence and disrupts cytoskeleton to facilitate ER targeting. ② Senescent tumor cell membrane–coated nanoparticles (B@SNP) exhibit more efficient ER targeting and induce intense ER stress compared with normal ones (B@NNP). ③ Tumor cells internalize B@SNP-derived MHC-I, present it on surface to initiate immune responses. ④ ER stress- and senescence-mediated antitumor immunity remodel the immune microenvironment. The illustration was created in BioRender. Tao, J. (2026) https://BioRender.com/o92m76p.

## RESULTS

### Physiological differences between senescent and normal tumor cells

Classic senescence-inducing drugs palbociclib (Pal) and ribociclib (Rib) were selected to induce senescence in mouse breast cancer 4T1 cells (*22*). Cells were induced with 1, 5, and 10 μM inducers for 24 hours, followed by detection of cell cycle arrest and senescence-associated β-galactosidase (SA-β-gal). Both senescence inducers triggered senescent cell phenotypes at high concentrations, accompanied by significant G_0_/G_1_ phase arrest and SA-β-gal accumulation (fig. S1, A and C). However, cytotoxicity assay revealed that high-concentration of Pal (above 5 μM) caused obvious cytotoxicity, whereas Rib showed no obvious cytotoxicity below 10 μM. Therefore, 10 μM of Rib was selected for subsequent senescence induction (fig. S1B).

Subsequently, we systematically evaluated the physiological disparities between normal (N) and senescent (S) 4T1 cells. First, scanning electron microscopy (SEM) was used to observe the morphology of normal and senescent 4T1 cells. Normal 4T1 cells exhibited a three-dimensional architecture with well-developed filopodia and lamellipodia, whereas senescent cells displayed increased volume, flattened morphology, reduced filopodia number and length, and collapsed lamellipodia (Fig. 2A). Given the intimate association between pseudopodia formation and cytoskeletal rearrangement, we examined alterations in cytoskeletal architecture during senescence transition. Normal 4T1 cells were characterized by a dense cytoskeletal network featuring well-organized stress fibers, lamellipodia, and filopodia. Conversely, senescent cells demonstrated a disorganized cytoskeleton, diminished actin levels, and failed to assemble stress fibers or pseudopodia (Fig. 2B), consistent with the SEM observations. Next, the image of SDS–polyacrylamide gel electrophoresis (SDS-PAGE) showed that senescent cell membranes expressed higher levels of multiple proteins than normal cell membranes in the range of 25 to 250 kDa (fig. S2). Proteomic analysis showed that senescent cell membranes contained 167 kinds of significantly up-regulated and 64 kinds of significantly down-regulated proteins compared to normal cell membranes (Fig. 2, C and D, and fig. S3). Gene Ontology analysis revealed that among the top 20 enriched protein categories in senescent cell membranes, over half were associated with ER membrane organization, vesicle trafficking, and other related cellular processes (Fig. 2E). Vesicle-associated membrane proteins (VAMPs) have been reported to primarily mediate the ER targeting of cell membrane–coated nanoparticles (*19*). Heatmap analysis of caveolins, VAMPs, and vesicle trafficking accessory proteins demonstrated their remarkable up-regulation in senescent cell membranes (Fig. 2F). In addition, MHC-I molecules involved in antigen presentation were remarkably increased in senescent cell membranes (fig. S4).

**Fig. 2.**
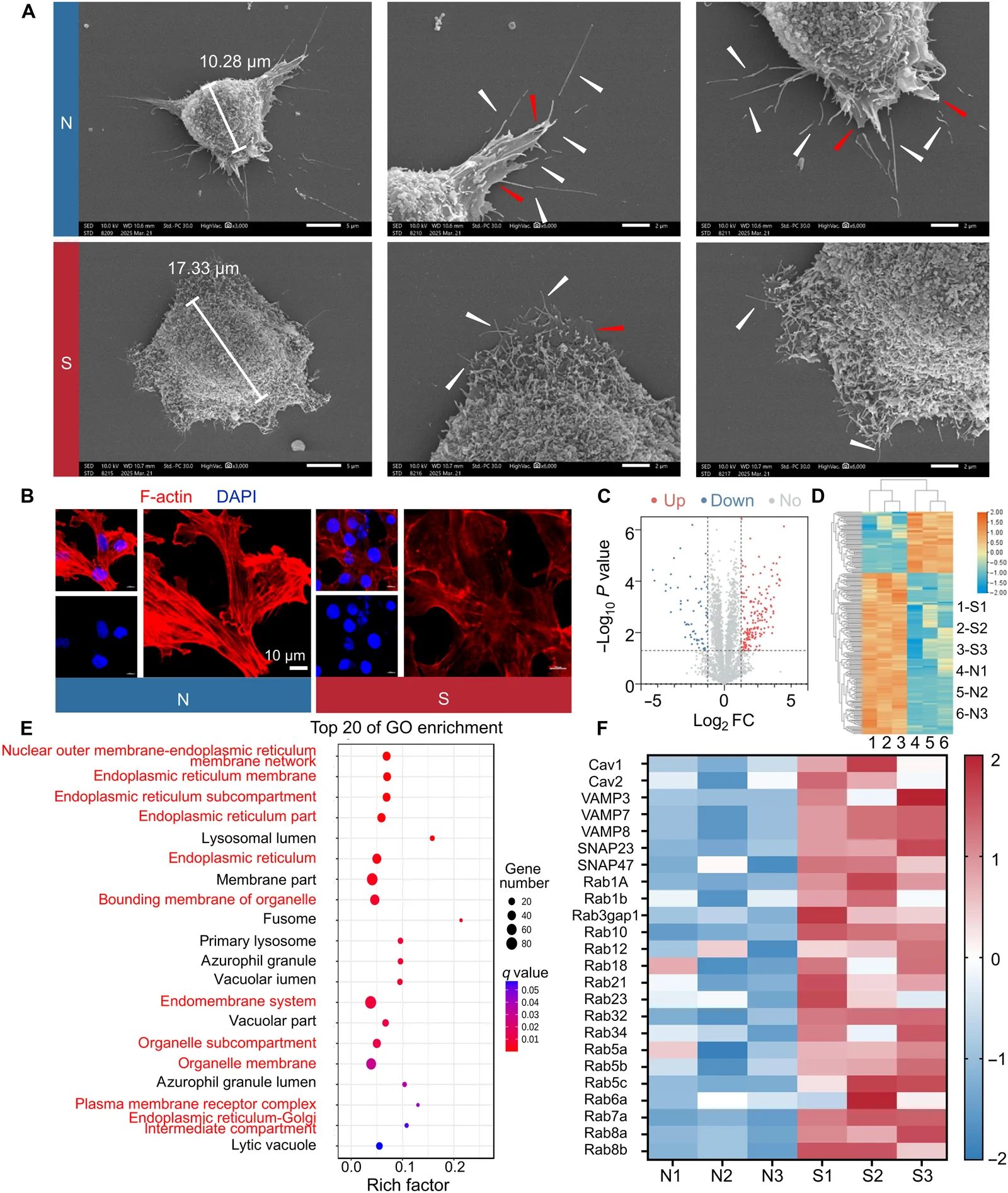
Physiological differences between senescent and normal tumor cells. (**A**) SEM images of the morphology of normal and senescent 4T1 cells. Scale bars, 5 and 2 μm. White arrows indicate filopodia, and red arrows indicate lamellipodia. (**B**) Confocal laser scanning microscopy (CLSM) images of cytoskeleton in normal and senescent 4T1 cells. Scale bar, 10 μm. Volcano plot (**C**) and heatmap analysis (**D**) of proteomics data comparing normal and senescent 4T1 cell membranes. (**E**) Gene Ontology (GO) analysis of the top 20 significantly enriched proteins on senescent 4T1 cell membranes. (**F**) Heatmap analysis of caveolins, VAMPs, and vesicle trafficking accessory proteins on normal and senescent 4T1 cell membranes. The experiment in (B) was repeated twice independently with similar results.

These findings underscore profound physiological differences between senescent and normal 4T1 cells. Disruption of the pseudopodia in senescent cells implies that inducing tumor cell senescence could impede metastatic potential (*23*, *24*). Conversely, elevated expression of caveolins (responsible for endocytosis), VAMPs (mediating ER targeting), and MHC-I molecules (key regulators of antigen presentation) in senescent cell membranes points to enhanced prospects for ER targeting and immunomodulation.

### Characterization of nanoparticles coated with tumor cell membranes

On the basis of the above findings, we engineered the senescent cell membrane–coated nanoparticles B@SNP, encapsulating brefeldin A (BFA) to trigger ER stress. Normal cell membrane–coated, BFA-loaded nanoparticles (B@NNP) were designed as a control in comparison with B@SNP.

First, 4T1 cells were induced to senesce via the aforementioned method, and both senescent and normal cell membranes were subsequently extracted (Fig. 3A). The drug-loaded poly(lactic-*co*-glycolic acid) (PLGA) nanoparticle cores were then prepared via nanoprecipitation, and, finally, the cell membranes were coated onto the nanoparticle surfaces using probe sonication (Fig. 3B). Malvern particle size analyzer showed that B@NNP measured 136.7 nm with a zeta potential of −28.3 mV, whereas B@SNP was slightly larger (146.0 nm) with a more negative zeta potential (−36.3 mV), likely attributable to the inherent properties of the different cell membranes (Fig. 3C). Both BFA-loaded nanoparticles exhibited favorable size dispersibility and a drug encapsulation efficiency (EE%) of approximately 70% (table S1). Both nanoparticles displayed spherical morphologies with clear cores, and distinct cell membrane structures were observable on their surfaces (Fig. 3D). The two nanoparticle formulations maintained stable physicochemical properties in phosphate-buffered saline (PBS, pH 7.4) and 10% fetal bovine serum (FBS), with less than 15% variation in particle size (fig. S5). Drug release profiles of the nanoparticles were investigated. Two BFA-loaded nanoparticles exhibited slow drug release over 72 hours, with a cumulative release rate of approximately 80% (Fig. 3E).

**Fig. 3.**
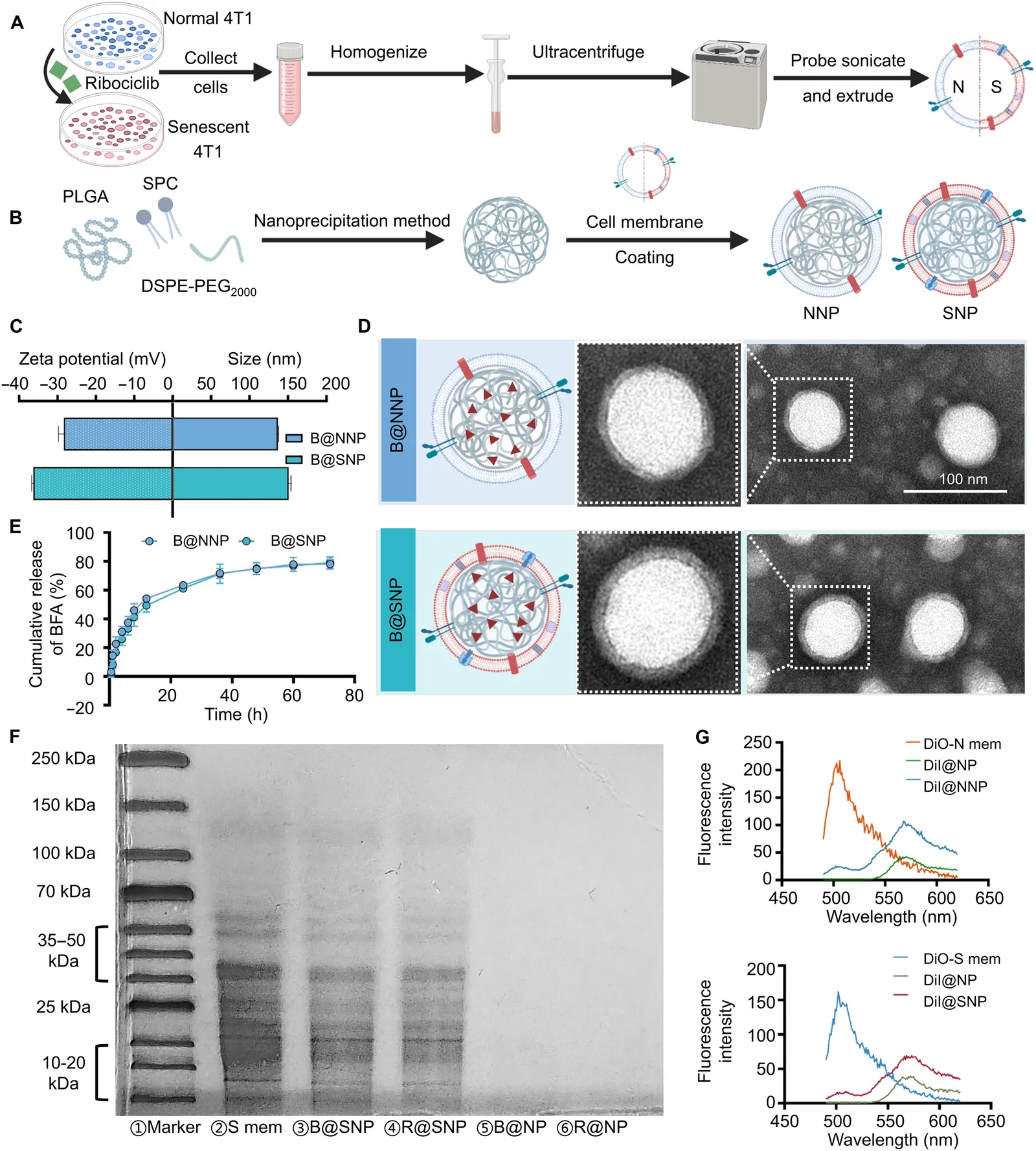
Characterization of nanoparticles coated with tumor cell membranes. (**A**) Flow chart for the extraction of normal or senescent 4T1 cell membranes. (**B**) Flow chart for preparation of membrane-coated nanoparticles. (**C**) Hydrodynamic diameter and zeta potential characterization of BFA-loaded nanoparticles. (**D**) Images of morphological analysis by TEM for BFA-loaded nanoparticles. Scale bar, 100 nm. (**E**) Release profile of BFA from B@NNP and B@SNP. (**F**) SDS-PAGE image of SNPs and senescent 4T1 cell membranes. (**G**) The FRET assay was performed to verify whether the cell membrane was successfully coated on the surface of the nanoparticles. The illustrations of (A), (B), and (D) were created in BioRender. Tao, J. (2026) https://BioRender.com/o92m76p. The experiment in (C) was repeated three times independently, and (E) to (G) were repeated twice independently with similar results. h, hours.

SDS-PAGE was used to confirm the coating of cell membranes on the surfaces of nanoparticles. The results demonstrated that membrane-coated nanoparticles retained the complete protein profiles of the cell membranes, while the nanoparticle cores showed no obvious protein bands (Fig. 3F). Fluorescence resonance energy transfer (FRET) experiments further confirmed that the distance between the cell membrane and the core of PLGA nanoparticles did not exceed 10 nm (Fig. 3G). Integrating data from particle size and zeta potential measurements, transmission electron microscope (TEM) imaging, protein profiling, and FRET assay, we confirmed the successful functionalization of nanoparticles with cell membranes.

### ER targeting and retention of tumor cell membrane-coated nanoparticles

Subsequently, we evaluated the cellular uptake efficiency and ER targeting specificity of the two membrane-coated nanoparticles in normal and senescent 4T1 cells. As shown in Fig. 4A, in both normal and senescent 4T1 cells, senescent tumor cell membrane–coated nanoparticles (SNP) were internalized significantly more efficiently than normal membrane–coated nanoparticles (NNP). In addition, SNP exhibited higher uptake in senescent cells compared with normal cells. The internalization mechanisms of different nanoparticles in normal or senescent cells were further explored (Fig. 4B). NNP entered cells via clathrin- and caveolin-mediated pathways, while SNP, owing to changes in surface physicochemical properties, primarily entered cells via caveolin-mediated endocytosis. Inducing senescence in recipient cells enhanced caveolin-dependent uptake, suggesting that marked up-regulation of caveolins in senescent cell membranes mediates the enhanced uptake, as observed in previous proteomics results.

**Fig. 4.**
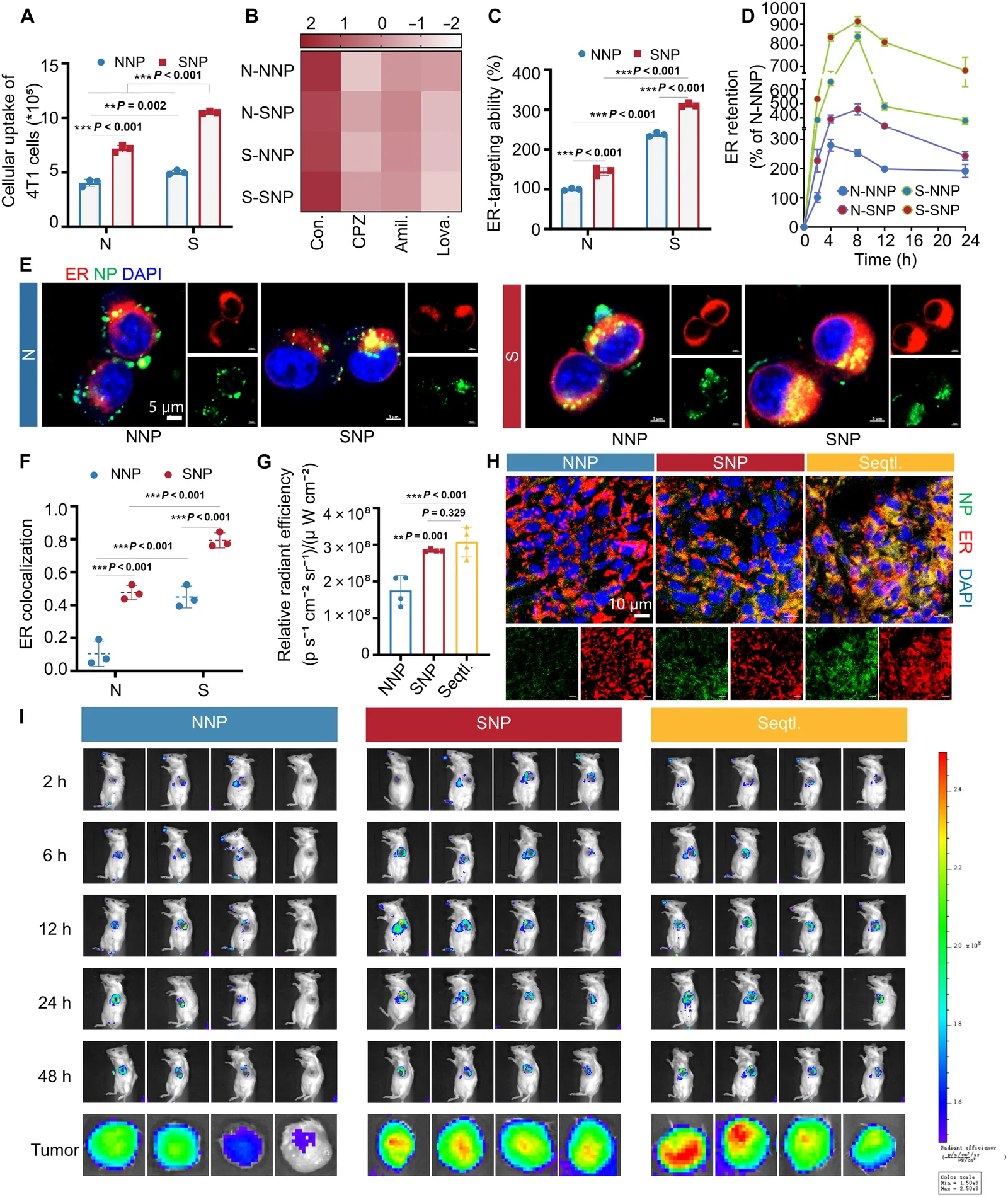
ER targeting and retention of tumor cell membrane–coated nanoparticles. (**A**) The uptake of two membrane-coated nanoparticles on normal or senescent 4T1 cells. (**B**) Investigation of the internalization mechanisms of two tumor membrane-coated nanoparticles on normal or senescent 4T1 cells. Assessment of ER targeting efficiency (**C**) and retention (**D**) of two membrane-coated nanoparticles on normal or senescent 4T1 cells. (**E**) CLSM images of colocalization between nanoparticles and ER of 4T1 cells. Scale bar, 5 μm. Red fluorescence indicates ER, green indicates nanoparticles, and blue indicates the nucleus. (**F**) The colocalization coefficient of ER and nanoparticles. (**G**) Semiquantitative analysis of membrane-coated nanoparticles accumulation at tumor sites in mice. (**H**) Images of colocalization between nanoparticles and ER of tumor cells in mice at 48 hours after intravenous tail-vein nanoparticle administration. Scale bar, 10 μm. Red fluorescence indicates ER, green indicates nanoparticles, and blue indicates the nucleus. (**I**) Accumulation of membrane-coated nanoparticles in mouse tumors at different time points after intravenous tail-vein nanoparticle administration. The experiments in (A) to (C) were repeated three times independently, and (D) to (F) were repeated twice independently with similar results. Data are presented as means ± SD, with *n* = 3. Statistical significance (*P* < 0.05) was determined by one-way analysis of variance (ANOVA) test.

We then examined the ER targeting and retention abilities of the nanoparticles. A pattern similar to cellular uptake was observed: SNP exhibited more efficient ER targeting and retention than NNP in both normal and senescent 4T1 cells. Moreover, for SNP, ER targeting efficiency and retention were significantly higher in senescent cells than in normal cells (Fig. 4, C to F). In vivo studies in orthotopic tumor–bearing mice further assessed tumor targeting and ER delivery performance of the membrane-coated nanoparticles (Fig. 4, G to I, and fig. S6). Consistent with in vitro results, SNP accumulated more in tumor tissues and targeted the ER. Notably, inducing tumor cell senescence markedly enhanced nanoparticle-ER colocalization, further demonstrating that senescence induction promotes ER targeting.

Compared with the diversified targeting strategies for organelles such as mitochondria and nuclei, current strategies for ER-targeted stress induction are primarily limited to ligand modification, including small molecules and peptides with ER-targeting properties. However, these strategies face multiple bottlenecks: (i) Difficulty in targeting: ER has complex structures and functions, with few exploitable targeting features; its distribution within the cytoskeletal network adds delivery challenges as drugs must cross this barrier. (ii) Limited retention: The rapid intracellular dynamics of ER impede efficient drug retention within the organelle. (iii) Proneness to compensation: ER stress readily activates compensatory pathways, weakening antimetastatic effects. These bottlenecks have rendered the development of ER-targeted drug delivery systems particularly challenging. Key challenges include enhancing drug accumulation in the ER, prolonging retention time, and blocking compensatory responses simultaneously. Senescent tumor cell membrane-coated nanoparticles demonstrated superior ER accumulation and sustained retention in 4T1 cells versus normal control group, effectively overcoming the dual bottlenecks of ER targeting and retention.

### Mechanisms of senescent cell membrane nanoparticles boosting ER targeting efficiency

After validating the superiority of inducing tumor cell senescence and using senescent cell membranes for ER delivery, we investigated the mechanisms underlying the enhancement of ER targeting on tumor cells. First, we compared the surface properties of drug-free NNP and SNP. Morphologically, SNP showed a significantly thicker membrane layer (18.7 versus 9.9 nm for NNP) as shown in Fig. 5A and fig. S7. Meanwhile, SNP exhibited a larger diameter and more negative zeta potential than NNP (Fig. 5, B and C). In addition, Young’s modulus measurements showed that SNP had a significantly higher stiffness than NNP (Fig. 5D). Since tumor cells preferentially internalize stiffer nanoparticles in the presence of receptor-ligand recognition, this may enhance the uptake of SNP (*25*). We hypothesized that these differences in surface properties are linked to the increased protein content and cholesterol levels in senescent tumor cell membranes (*26*). Elevated cholesterol content can enhance stiffness, while proteins influence the zeta potential of membrane-coated nanoparticles.

**Fig. 5.**
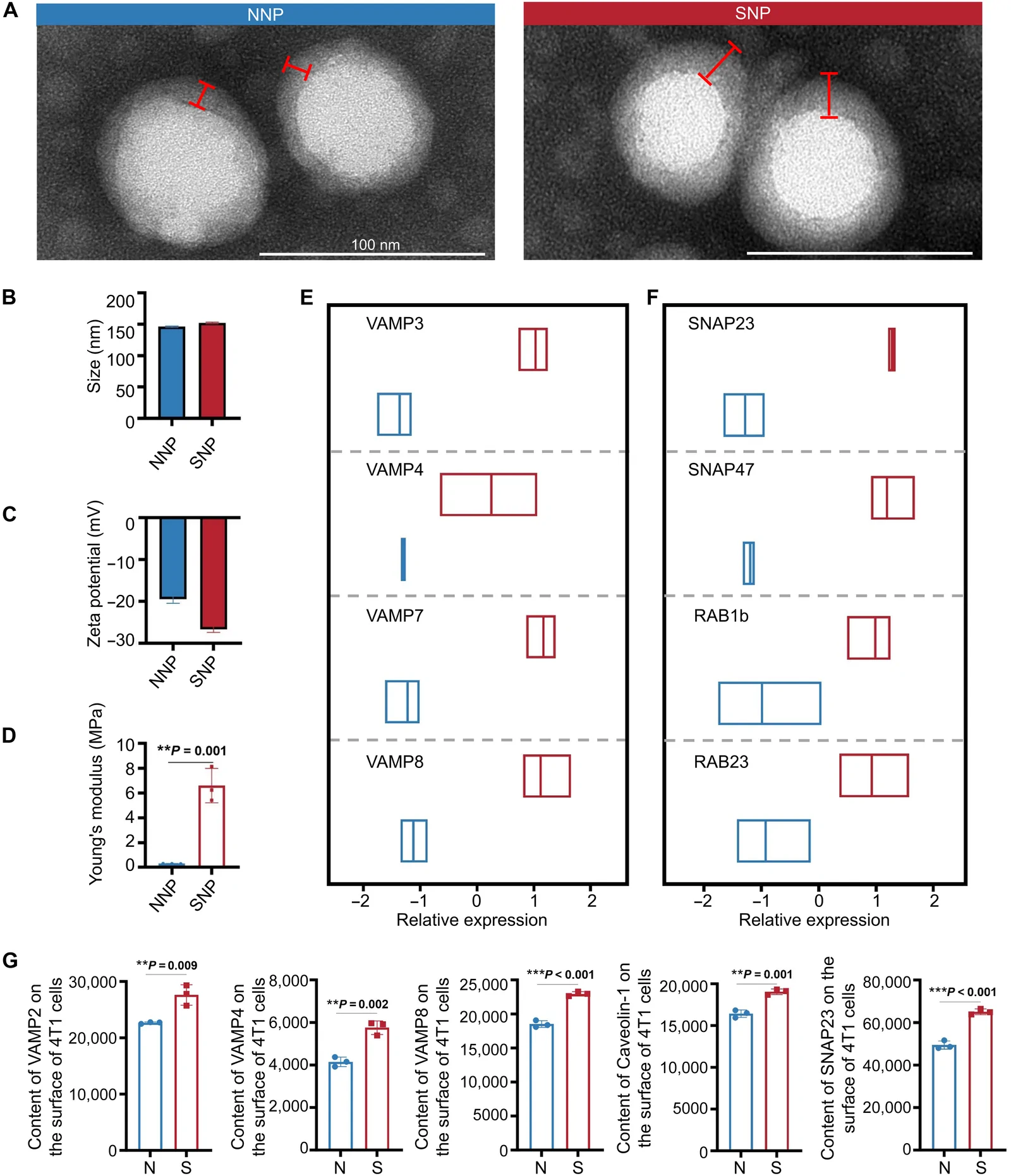
Mechanisms of senescent cell membrane nanoparticles boosting ER targeting efficiency. (**A**) The morphology of drug-free NNP and SNP observed by TEM. Scale bar, 100 nm. Size (**B**), zeta potential (**C**), and Young’s modulus (**D**) of NNP and SNP. Expression of VAMPs (**E**) and trafficking accessory proteins (**F**) on normal and senescent 4T1 cell membranes via proteomic analysis. (**G**) The expression of VAMP2, VAMP4, VAMP8, caveolin-1, and SNAP23 on normal and senescent tumor cell membranes by flow cytometry. The experiments in (B) to (C) were repeated three times independently with similar results. Data are presented as means ± SD, with *n* = 3. Statistical significance (*P* < 0.05) was determined by Student’s *t* test.

Furthermore, proteomic analysis revealed up-regulated expression of VAMPS (VAMP3, VAMP4, etc.) and trafficking accessory proteins (SNAP23, RAB1b, etc.) that mediate ER targeting in senescent cell membranes (Fig. 5, E and F). Flow cytometric quantification further confirmed this observation (Fig. 5G). These findings elucidate how senescent cell membranes promote ER targeting. Concurrently, the ER molecular chaperone BiP (binding immunoglobulin protein) was significantly up-regulated on the surface of senescent tumor cells (fig. S8). The C-terminal ER retention signal of BiP, KDEL, which binds to KDEL receptors, could prevent nanoparticles from being transported to the Golgi apparatus (*27*). Therefore, nanoparticles can be specifically enriched and retained in the ER lumen.

### Senescent tumor membrane nanoparticles initiate immunity via MHC-I delivery

Studies have shown that senescent tumor cells up-regulate MHC-I molecule expression, which can promote dendritic cell activation and maturation (*28*). Since MHC-I is expressed on the cell membrane, we hypothesized whether senescent tumor cell membranes could also retain the immune-activating potential of senescent tumor cells.

First, we treated 4T1 cells with NNP or SNP and found that both nanoparticles increased MHC-I levels in 4T1 cells, with SNP exhibiting a stronger enhancing effect. We then examined immune-related cytokine levels and observed consistent results: SNP more potently promoted the secretion of IFN-γ, IL-1β, and TNF-α by 4T1 cells (Fig. 6A). MHC-I molecules and cytokines are involved in immune microenvironment remodeling, antigen presentation, and immune cell activation, suggesting that senescent cell membranes may have vaccine-like potential.

**Fig. 6.**
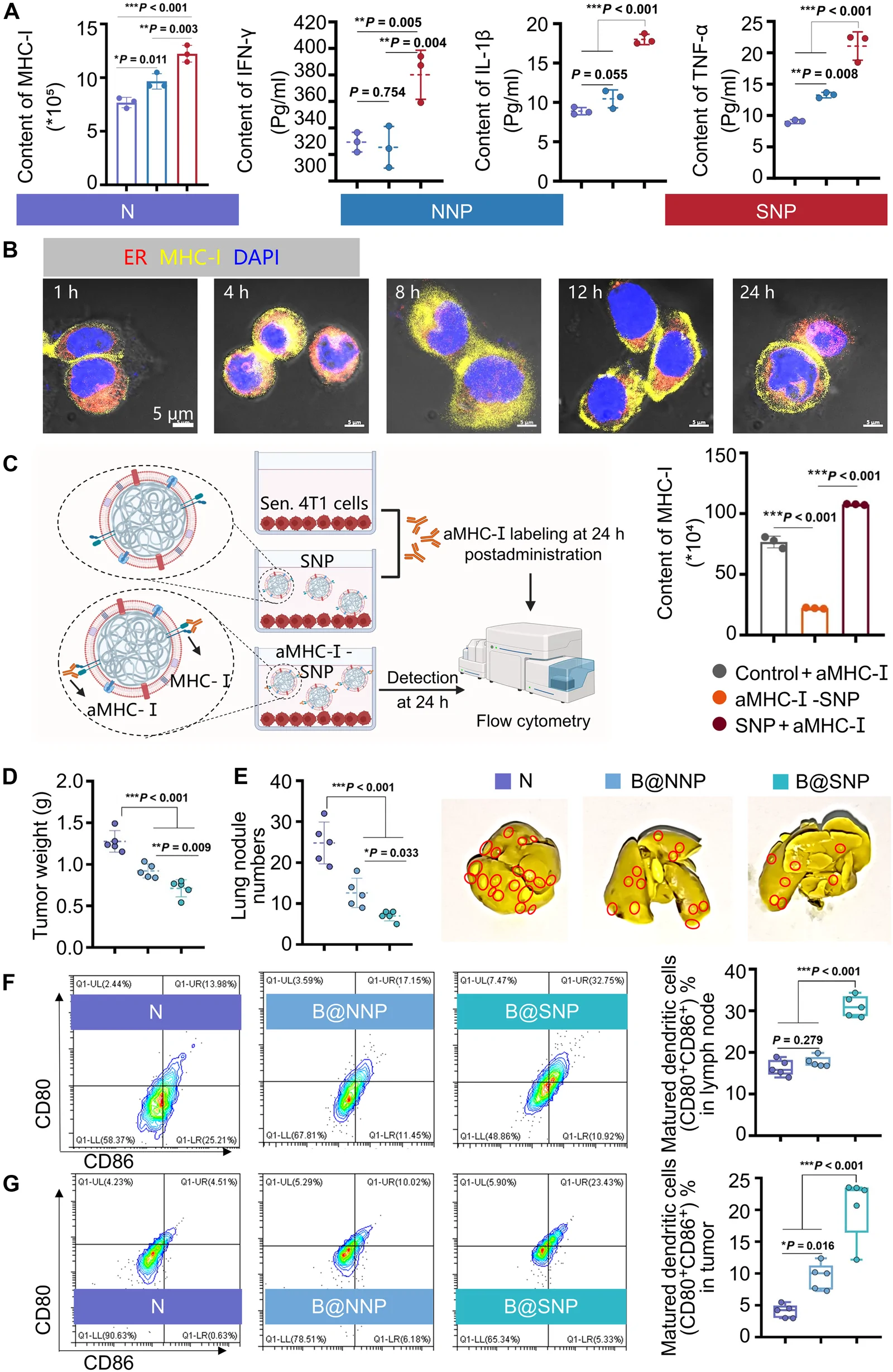
Senescent tumor membrane nanoparticles initiate immunity via MHC-I delivery. (**A**) Expression of MHC-I molecules, secretion of IFN-γ, IL-1β, and TNF-α in 4T1 cells after treatment with NNP or SNP. (**B**) CLSM images of exogenous MHC-I molecule delivery from SNP to 4T1 cells. Scale bar, 5 μm. Red fluorescence indicates ER, yellow indicates MHC-I, and blue indicates the nucleus. (**C**) Schematic and flow cytometric investigation into the source of up-regulated MHC-I molecules in 4T1 cells. (**D**) Tumor weight of mice in all groups at 18 days postadministration. (**E**) Statistical results and images of lung nodules. Dendritic cell infiltration in lymph nodes (**F**) and tumor tissues (**G**). The illustration of (C) was created in BioRender. Tao, J. (2026) https://BioRender.com/o92m76p. The experiments in (B) and (C) were repeated twice independently with similar results. Data are presented as means ± SD, with *n* = 3 in vitro, *n* = 5 in vivo. Statistical significance (*P* < 0.05) was determined by one-way ANOVA test.

To determine whether the increased MHC-I levels induced by senescent cell membranes originated from endogenous tumor cell expression or exogenous supply from the cell membranes themselves, we used confocal microscopy and flow cytometry for investigation. Confocal imaging revealed that exogenous MHC-I molecules on SNP initially colocalized with ER upon internalization, followed by translocation from ER to the cell membrane surface (Fig. 6B). This process mimics the transport of endogenous MHC-I molecules in tumor cells, demonstrating that SNP can deliver exogenous MHC-I molecules on their membranes to tumor cells, anchor them to the cell surface, and thereby recruit immune cells. Flow cytometry results further validated this finding: 4T1 cells directly “acquired” exogenous MHC-I molecules from SNP (labeled with MHC-I antibodies) rather than up-regulating endogenous MHC-I expression via intracellular signaling pathways (Fig. 6C).

Furthermore, given that ER stress elicits a robust immune response, we encapsulated BFA, an ER stress inducer, within normal or senescent tumor membrane-coated nanoparticles (B@NNP or B@SNP). This design aims to use senescent tumor cell membranes to deliver BFA to ER, thereby inducing ER stress and triggering antitumor immune responses. Meanwhile, the capacity of normal and senescent tumor cell membranes to regulate the tumor immune microenvironment has been compared. We evaluated the immune-regulatory capacity of B@NNP and B@SNP in a murine tumor model (fig. S9). Compared with B@NNP, B@SNP exhibited higher tumor growth inhibition and lung metastasis suppression rates (Fig. 6, D and E, and fig. S10). Analysis of immune-related parameters showed that B@SNP effectively increased MHC-I levels on tumor cell surfaces, promoted secretion of IFN-γ, IL-1β, and TNF-α (fig. S11). Meanwhile, the infiltration of mature dendritic cells in lymph nodes and tumor tissues was significantly enhanced by B@SNP (Fig. 6, F and G). The above results demonstrate that B@SNP can deliver encapsulated ER stress inducer BFA and membrane-surface MHC-I molecules in vivo, thereby effectively remodeling the immune microenvironment, promoting antigen presentation, and activating immune cells.

### Efficacy of the sequential strategy against tumor metastasis in vitro

Although senescent cell membranes enhanced the antitumor efficacy of ER-targeted nanoparticles, the tumor inhibition rate only reached 44.2%. Furthermore, lung metastasis remained unresolved, falling short of the desired therapeutic outcomes (Fig. 6, D and E). This may be due to the insufficient targeting ability of senescent tumor membrane–coated nanoparticles toward normal tumor cells, resulting in low drug accumulation. In addition, ER stress inducers alone may activate compensatory pathways, thus enabling tumor cells to evade apoptosis (*29*). Therefore, we adjusted our strategy: Before administering the ER stress inducer (B@SNP), we first induced tumor cells into a senescent phenotype using Rib-loaded nanoparticles coated with senescent tumor cell membranes (R@SNP). This pretreatment not only primes the ER of tumor cells into a sensitive state but also restricts their motility. Building on this senescent priming, delivering the ER stress inducer is expected to elicit comprehensive and robust ER stress, circumvent compensatory responses, and thereby achieve complete elimination of tumor cells. Characterization of R@SNP confirmed a well-defined spherical morphology (126.5 nm, −30.1 mV), along with favorable drug release profiles and excellent stability (Fig. 7, A and B, fig. S5, and table S1). In vivo biodistribution experiments revealed that SNP achieved maximal tumor accumulation at 24 hours, while in vitro incubation with Rib for 24 hours could trigger significant cellular senescence. Thus, a 24-hour interval was adopted for sequential drug delivery. To validate the rationality of the dosing interval, we first quantified intratumoral accumulation of both agents after sequential administration in tumor-bearing mice; the corresponding results are shown in fig. S12. Rib peaked in tumor tissue at 24 hours and underwent substantial clearance by 48 hours. BFA, delivered 24 hours post-Rib, displayed a similar distribution profile: Its tumor concentration peaked at 24 hours with markedly prolonged retention, and full elimination from tumor tissue was not observed even 48 hours after BFA injection. This pattern can likely be attributed to senescence triggered by Rib, which extends the retention of ER-targeted nanoparticles (B@SNP) within the ER. In contrast, a shorter time gap would block sufficient senescence induction by Rib, thereby impairing the ER-targeting efficiency of subsequently injected B@SNP. Conversely, an excessively prolonged interval leads to sustained senescent cell populations that secrete metastasis-promoting senescence-associated secretory phenotype (SASP) cytokines (IL-6 and IL-10) and accelerate tumor metastatic progression (*30*, *31*). Collectively, a 24-hour interval was selected as the optimal schedule for sequential drug administration.

**Fig. 7.**
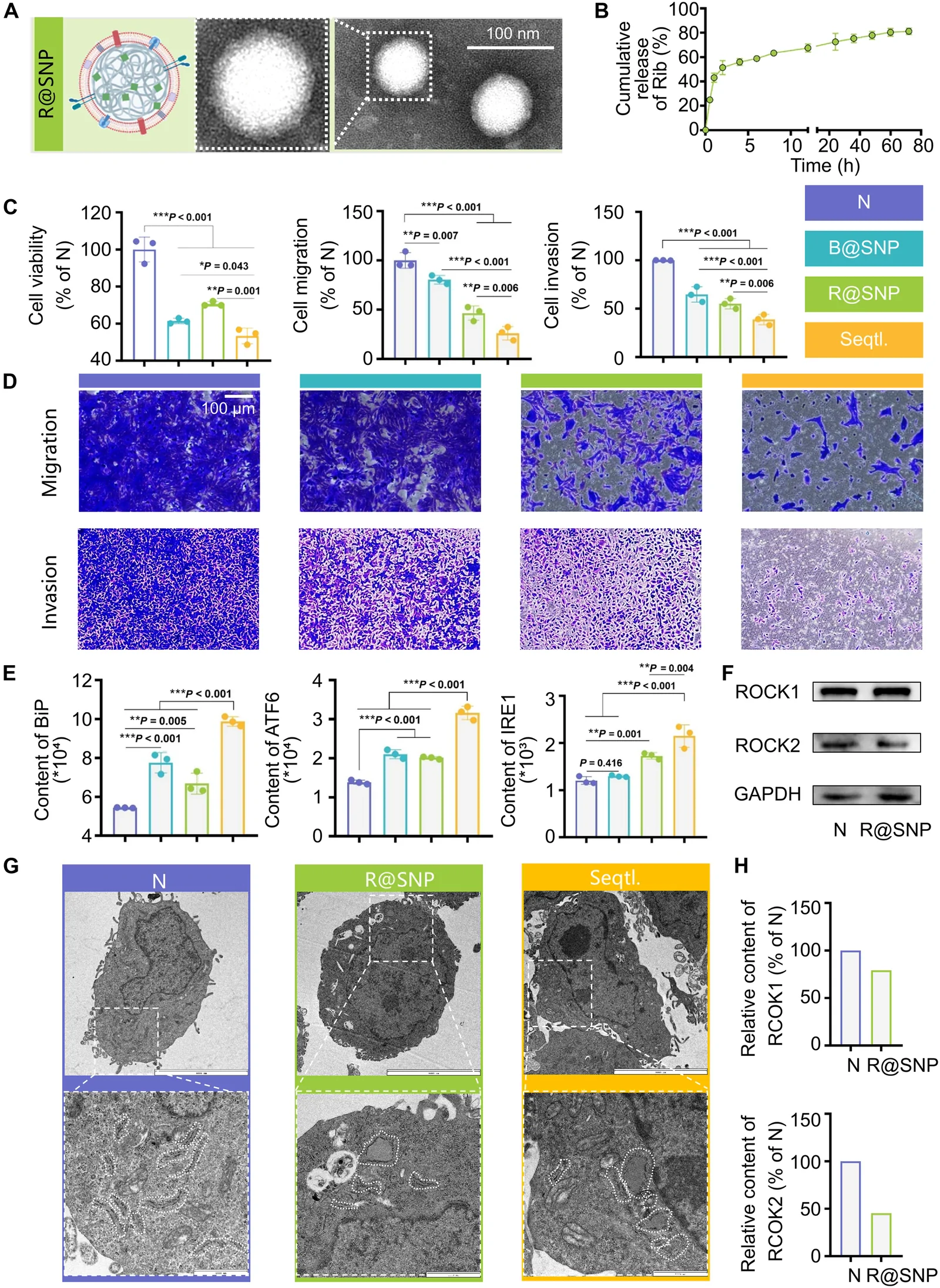
Efficacy of the sequential strategy against tumor metastasis in vitro. (**A**) TEM images of R@SNP. Scale bar, 100 nm. (**B**) Release profile of Rib from R@SNP. (**C**) Cell viability, cell migration, and cell invasion evaluation postadministration of various drug formulations. (**D**) Microscopic images of cell migration and invasion. Scale bar, 100 μm. (**E**) Quantitative flow cytometry analysis of BiP, ATF6, and IRE1 levels. (**F**) WB analysis of ROCK proteins. N for normal 4T1 cells. (**G**) Morphological observation of ER by TEM. Scale bar, 1000 and 6000 nm. (**H**) Semiquantitative analysis of ROCK protein from WB. The illustration of (A) was created in BioRender. Tao, J. (2026) https://BioRender.com/o92m76p. The experiments in (B) to (E) were repeated twice independently with similar results. Data are presented as means ± SD, with *n* = 3. Statistical significance (*P* < 0.05) was determined by one-way ANOVA test.

Afterward, in vitro experiments using 4T1 cells validated the efficacy of this sequential strategy. First, cell viability assay in 4T1 cells showed that B@SNP significantly inhibited cell growth, attributed to ER stress–initiated programmed cell death. Inducing cell senescence by R@SNP significantly suppressed cell migration and invasion (Fig. 7, C and D). Mild antimetastatic effect was also observed after ER stress. The sequential group (senescence induction followed by ER targeting, Seqtl.) showed the most profound suppression of cell viability, migration, and invasion, highlighting the efficacy against tumor progression and metastasis.

To explore the antitumor mechanism, we analyzed ER stress–associated markers. The ER stress pathway is highly intricate, involving multiple signaling cascades mediated by key molecules such as BiP, ATF6, and IRE1 (*32*). B@SNP only activated the BiP and ATF6 pathways, suggesting that tumor cells may compensate through the IRE1 pathway to evade apoptosis during ER stress. Notably, inducing cell senescence by R@SNP-sensitized ER by regulating the IRE1 pathway. The Seqtl. group, which first induced cellular senescence and then delivered ER stress inducer, fully activated multiple ER stress pathways, leading to profound and robust ER stress (Fig. 7E). Morphological observation of ER by TEM also corroborated this finding. ER in normal 4T1 cells exhibited a slender, thread-like morphology. Notably, in senescent 4T1 cells, segments of ER exhibited pronounced dilation with increased lumenal volume, demonstrating that induction of tumor cell senescence sensitizes ER to stress. In the Seqtl. group receiving ER stress inducer after senescence induction, extensive ER dilation was observed, indicating the highest level of ER stress (Fig. 7G). Consequently, the bottleneck of ER delivery—proneness to compensation—has also been addressed.

In addition, we investigated the antimetastasis mechanism. Western blot (WB) and flow cytometry results showed that senescence induction reduced the expression of ROCK1 and ROCK2 in tumor cells (Fig. 7, F and H). ROCK proteins mediate cytoskeletal rearrangement; their down-regulation disrupts cytoskeletal architecture, impairing stress fiber and pseudopodia formation—consistent with previous SEM and confocal microscopy observations (*33*). Thus, the sequential strategy suppresses tumor progression by activating ER stress pathways and blocking the Rho/ROCK axis.

### Efficacy of the sequential strategy against tumor metastasis in vivo

Antitumor metastasis efficacy was also evaluated in orthotopic 4T1 tumor–bearing mice (Fig. 8A). We found that ER stress inducer (B@SNP) effectively inhibited orthotopic tumor growth, while the senescence inducer (R@SNP) reduced the number of lung nodules to fewer than 10. The simultaneous administration group (Simul.) exhibited tumor growth inhibition and lung nodule reduction comparable to those of B@SNP or R@SNP, likely due to insufficient tumor accumulation of nanoparticles. In contrast, the sequential administration group (Seqtl.) achieved the highest tumor growth inhibition rate (80.2%) and the fewest number of lung nodules (zero to three) (Fig. 8, B to E). Hematoxylin and eosin staining images of lung tissues from tumor-bearing mice further confirmed these results (fig. S13).

**Fig. 8.**
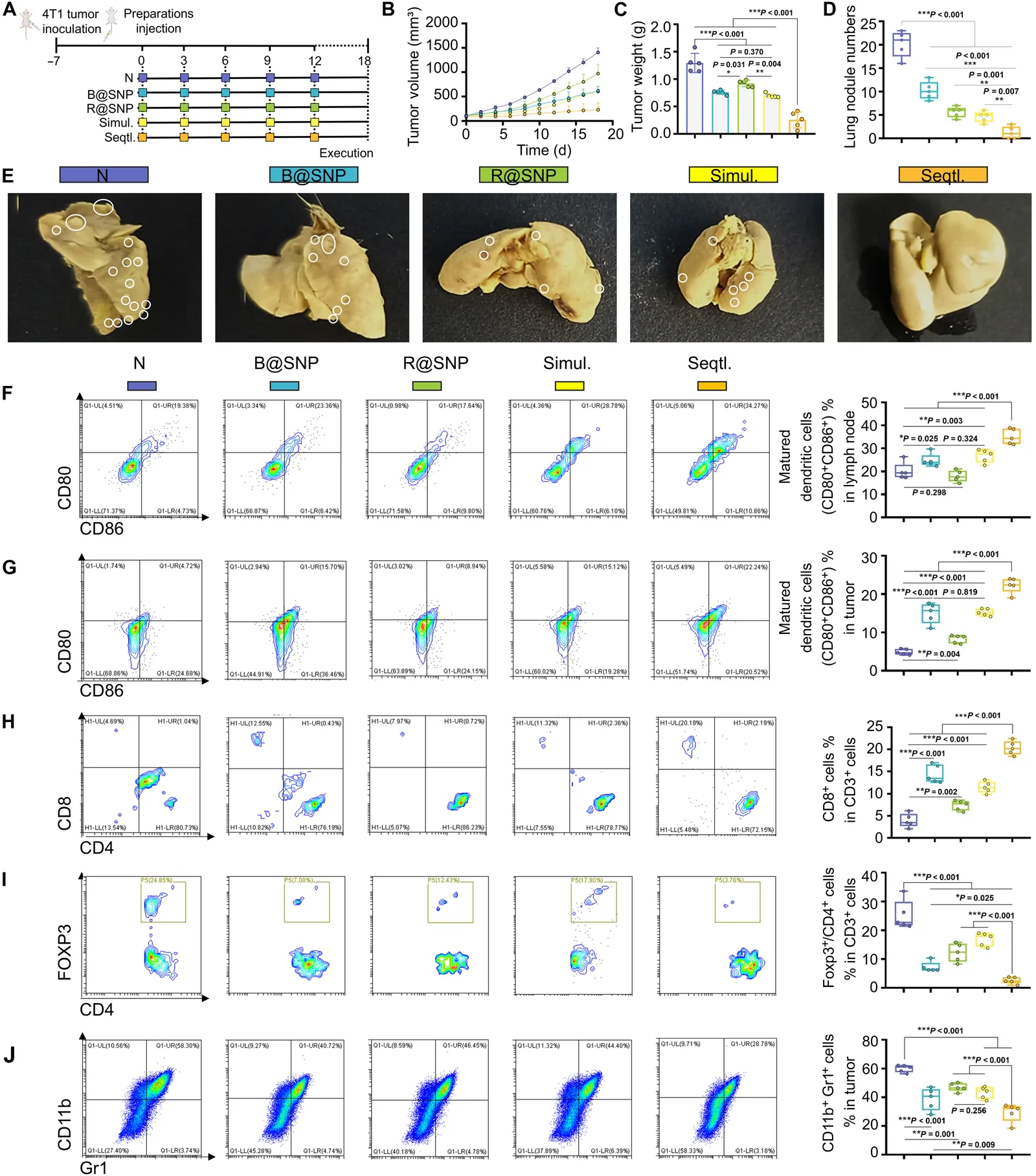
Efficacy of the sequential strategy against tumor metastasis in vivo. (**A**) Administration regimen of the sequential therapeutic strategy in vivo. (**B**) Curve chart for tumor volume monitoring. (**C**) Tumor weight of mice at 18 days postadministration. (**D**) The number of lung nodules 18 days after administration. (**E**) Image acquisition of lung nodule. Infiltration of dendritic cells in lymph nodes (**F**) and tumor tissues (**G**), CD8^+^ T cells (**H**) and Tregs (**I**) and MDSCs (**J**) in tumor tissues. The illustration of (A) was created in BioRender. Tao, J. (2026) https://BioRender.com/o92m76p. Data are presented as means ± SD, with *n* = 5 in vivo. Statistical significance (*P* < 0.05) was determined by one-way ANOVA test.

Infiltration of immune cells revealed that both single-drug groups increased the abundance of dendritic cells in lymph nodes and tumor tissues, augmented intertumoral CD8^+^ T cells recruitment, and reduced infiltration of immunosuppressive cells [regulatory T (T_reg_) cells and myeloid-derived suppressor cells (MDSCs)]. The Simul. group showed similar effects to B@SNP or R@SNP, whereas the Seqtl. group exhibited the highest levels of immune cell infiltration (Fig. 8, F to J). These findings suggest that the sequential strategy potently drives immune cell recruitment, augments antigen-presenting cell activity, and enhances functional immune cell infiltration, ultimately eliminating tumor cells.

### Mechanisms of the sequential strategy against tumor metastasis in vivo

Building on the promising antitumor metastasis efficacy of the sequential strategy, we explored the underlying mechanisms in vivo. Consistent with the expression of ER stress markers in vitro, the Seqtl. group simultaneously activated BiP, ATF6, and IRE1 signaling pathways, inducing comprehensive and sustained ER stress (Fig. 9, A to C). Expression of ICD markers was also evaluated after ER stress. As shown in Fig. 9 (D and E) and fig. S14, compared with the B@SNP or R@SNP, the Seqtl. group significantly promoted cell surface exposure of calreticulin (CRT) and secretion of high-mobility group box 1 protein (HMGB1), accompanied by reduced intracellular HMGB1 levels. These findings indicate that sustained ER stress could trigger ICD, recruit immune cells, and activate systemic immune responses.

**Fig. 9.**
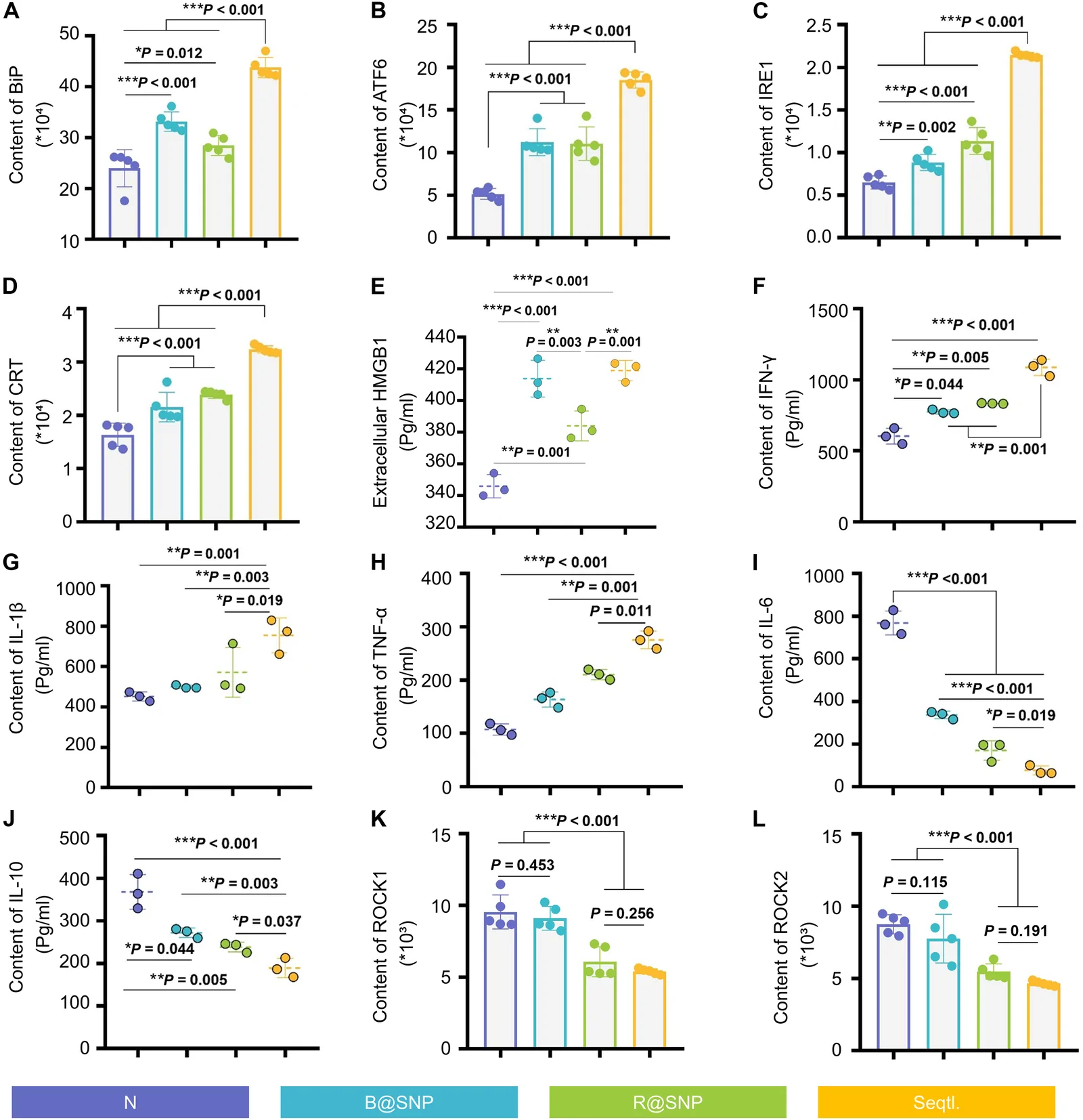
Mechanisms of the sequential strategy against tumor metastasis in vivo. Expression of BiP (**A**), ATF6 (**B**), and IRE1 (**C**) in tumor cells in vivo. (**D**) Exposure of CRT on the tumor cell surface. (**E**) Secretion of extracellular HMGB1 in tumor tissues. The content of immune-related cytokines IFN-γ (**F**), IL-1β (**G**), and TNF-α (**H**) in tumor tissues. Levels of IL-6 (**I**) and IL-10 (**J**) at tumor sites. Expression of ROCK1 (**K**) and ROCK2 (**L**) in tumor cells. Data are presented as means ± SD, with *n* = 3 or 5 in vivo. Statistical significance (*P* < 0.05) was determined by one-way ANOVA test.

Moreover, we analyzed the secretion of SASP implicated in immune regulation and tumor metastatic progression. The Seqtl. group exhibited the highest secretion levels of IFN-γ, IL-1β, and TNF-α, potentially mediating remodeling of immune microenvironment and activation of immune cells (Fig. 9, F to H) (*34*, *35*). Conversely, IL-6 acts as a key prometastatic cytokine, driving tumor cell migration and invasion through epithelial-mesenchymal transition (EMT) and inflammatory microenvironment modulation (*36*). Similarly, IL-10 orchestrates immunosuppression by dampening antigen-presenting cell function to promote tumor progression and metastatic cascade (*37*). Both cytokines were minimally expressed in the Seqtl. group (Fig. 9, I and J). Induction of tumor cell senescence represents a well-validated therapeutic strategy for enhancing the tumor immune microenvironment and achieving effective tumor growth control. However, tumor cell senescence extends beyond mere growth arrest; the prolonged presence of SASP poses potential risks, including immunosuppression, stimulation of EMT, and promotion of tumor migration and metastasis (*38*). Balancing this potential “double-edged sword”—leveraging cell senescence to enhance the immune microenvironment while avoiding long-term SASP-mediated adverse effects and achieving timely clearance of senescent cancer cells—warrants in-depth investigation. Our sequential strategy effectively harnesses beneficial SASP effects to regulate immune and metastatic microenvironments. Simultaneously, it mitigates SASP-promoted metastasis, thus optimizing therapeutic benefits while minimizing risks.

Last, the metastasis-related Rho/ROCKs signaling pathway was analyzed using flow cytometry. Notably, both R@SNP and Seqtl. groups significantly down-regulated ROCK1/ROCK2, leading to cytoskeletal disruption and subsequent tumor metastasis inhibition (Fig. 9, K and L).

Collectively, these results demonstrate the superior efficacy of the sequential strategy, which first induces cell senescence followed by ER stress induction. This sequential strategy maximizes ER stress, driving activation of ICD-mediated immune responses. During the sequential administration, the tumor immune and metastatic microenvironment were also remodeled through SASP. In addition, the strategy suppresses ROCK1/ROCK2-mediated cytoskeletal reorganization, a key step in tumor metastasis. Through the synergistic orchestration of these mechanisms, the strategy elicits profound suppression of tumor proliferation and metastatic dissemination.

### Safety assessment of the sequential strategy in vivo

Ultimately, the safety profile in vivo of the sequential strategy was systematically evaluated. Routine hematological analysis revealed that tumor-bearing mice, when compared to healthy control mice, exhibited decreased erythrocyte counts and significantly elevated leukocyte, platelet, neutrophil, monocyte, and lymphocyte counts, putatively attributed to the inflammatory tumor microenvironment (*39*). Following sequential administration, hematological parameters in the Seqtl. group demonstrated a trend toward normalization, approaching those of healthy control mice (Fig. 10, A to H). Serum biochemical analysis revealed no significant evidence of hepatic or renal toxicity in the Seqtl. group (Fig. 10, I to L). SA-β-gal staining of major accumulation sites (liver, spleen, and tumor) of SNP revealed no obvious positive staining (green) in the three organs of the saline group or the liver and spleen of the Seqtl. group. In contrast, tumor tissues from the Seqtl. group exhibited a marked number of SA-β-gal–positive cells with green staining, providing evidence for the selective induction of tumor cell senescence by the senescence inducer without affecting normal organs (Fig. 10M). In addition, hemolysis assays (fig. S15) and in vivo toxicity tests of nanoparticles (fig. S16) showed no hemolytic activity and no treatment-related mouse deaths. Collectively, these findings provide robust evidence for the in vivo safety profile of the sequential therapeutic strategy.

**Fig. 10.**
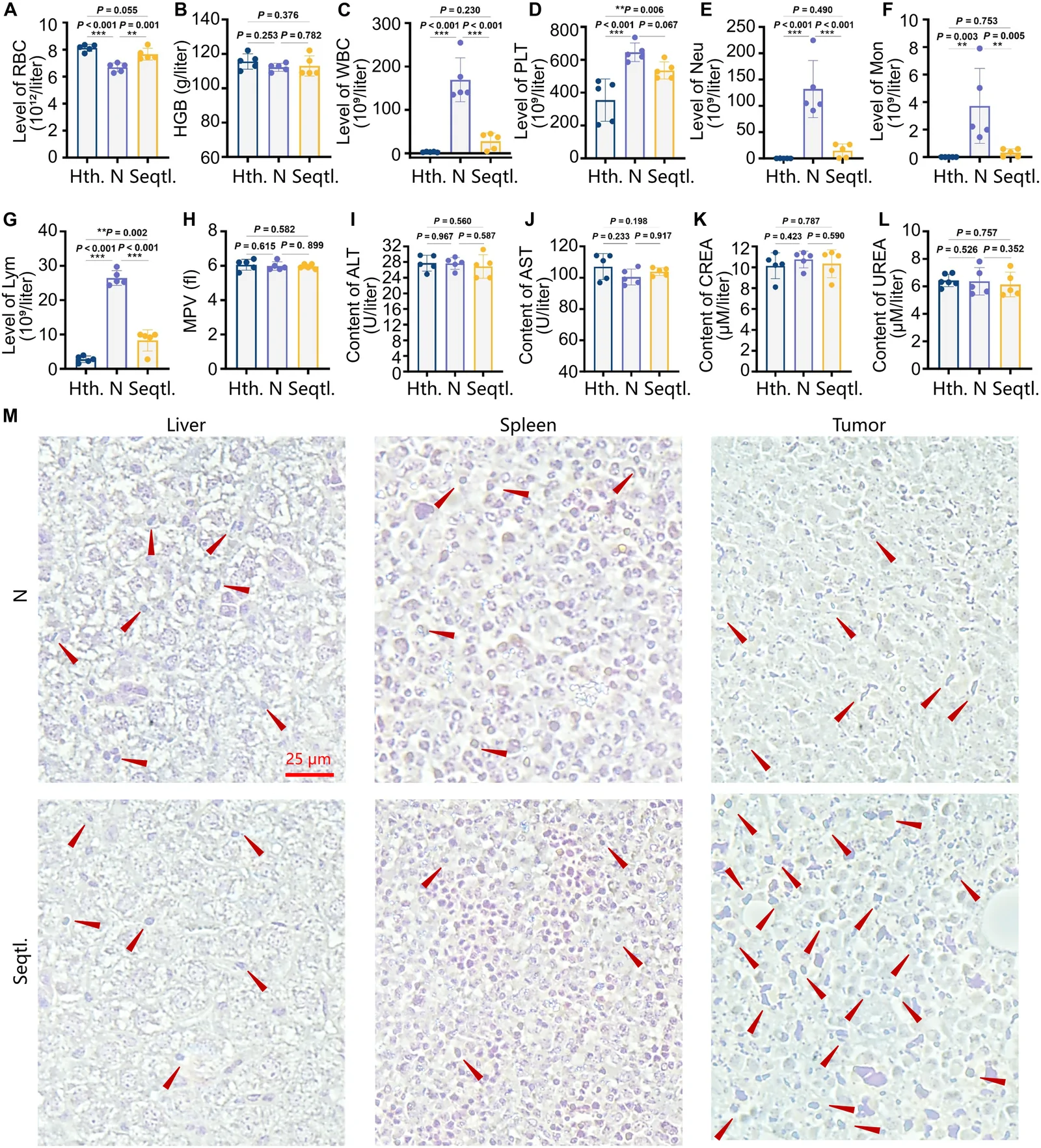
Safety assessment of the sequential strategy in vivo. (**A** to **H**) Routine hematological profiling of healthy control, saline-treated, and sequential treatment group mice. (**I** to **L**) Serum biochemical analysis of healthy control, saline-treated, and sequential treatment group mice. (**M**) Images of SA-β-Gal staining for the liver, spleen, and tumor tissues from group N (tumor-bearing mice treated with saline) and group Seqtl. (the sequential administration group, tumor-bearing mice treated with R@SNP, followed by B@SNP treatment at a 24-hour interval). The red arrows indicate senescent cells. Scale bar, 25 μm. Data are presented as means ± SD, with *n* = 5 in vivo. Statistical significance (*P* < 0.05) was determined by one-way ANOVA test.

## DISCUSSION

Cells at different life cycles exhibit unique physiological properties and are thus endowed with distinct homing characteristics (*40*–*43*). For instance, the natural spleen-homing property of senescent erythrocytes has been used to construct spleen-targeted drug delivery systems (*44*). Similarly, the bone marrow–homing ability of senescent neutrophils has been used to treat myeloma via a hitchhiking strategy (*45*). However, research on drug delivery systems based on the physiological differences of cells at different life cycles remains in the preliminary stage. Furthermore, the influence of cell membranes at different life cycles on subcellular delivery efficiency remains unexplored (*46*–*48*). As the core functional units of cells, subcellular structures play a central role in sustaining normal cellular activities. Their functional homeostasis and the underlying regulatory mechanisms represent a key research direction in tumor therapy (*49*, *50*). Therefore, ER serves as a critical target for tumor metastasis. Enhancing the targeting and accumulation of drugs in ER represents an effective strategy for the treatment of tumor metastasis.

Biomimetic drug delivery systems based on cell membrane coating have been reported for subcellular delivery (*20*, *21*). Our group previously demonstrated that tumor cell membrane–coated nanoparticles not only exhibit homing effects toward homologous tumors and high affinity for tumor cells but also enable specific accumulation in ER-Golgi apparatus membrane system during intracellular trafficking (*19*). Therefore, we performed further investigations based on these findings. We found that SNP exhibited more efficient ER targeting and retention than NNP in both normal and senescent 4T1 cells. Moreover, for SNP, ER-targeting efficiency and retention were markedly higher in senescent cells than in normal cells. By comparing the in vivo antimetastatic efficacy of B@NNP and B@SNP, we demonstrated that senescent cell membranes markedly enhanced the antimetastatic activity of nanoparticles by promoting their accumulation at tumor sites and modulating the tumor immune microenvironment.

Nevertheless, the low proportion of senescent cells in tumor tissues restricts the full homing effect of SNP (*51*). Moreover, the compensatory response induced by ER stress results in B@SNP alone failing to achieve optimal antimetastatic efficacy (*52*). Therefore, we first induced tumor cells into a senescent phenotype before the administration of ER-targeted nanoparticles. On one hand, this strategy enhances the accumulation of B@SNP at tumor sites to maximize its therapeutic efficacy; on the other hand, senescence induction can synergize with BFA to trigger intense ER stress, thereby blocking the compensatory pathways activated following ER stress. The senescence inducer (R@SNP) not only enhanced the ER targeting and retention of nanoparticles but also promoted ER stress and regulated the SASP. In synergy with ER stress–inducing nanoparticles (B@SNP), the sequential strategy reshaped the tumor immune microenvironment to enable tumor cell elimination. The tumor inhibition rate exceeded 80%, and the number of pulmonary nodules was nearly zero, indicating remarkable antimetastatic efficacy in 4T1 tumor–bearing mice.

In summary, our study addresses the critical challenges of insufficient ER targeting, limited retention, and prone compensation in tumor cells, filling the knowledge gap in the regulation of cell membrane life cycles for the design and development of drug delivery systems. Compared with other live cell–based hitchhiking strategies, our system not only enables efficient drug loading, stable and precise delivery, and sustained release, but also exhibits excellent in vivo biosafety (*41*, *42*). Although we achieved efficient ER-targeted delivery using senescent cell membranes, the standards for the extraction and purification of senescent tumor cell membranes still require further optimization. In addition, the patient tolerance of intravenously administered senescent cell membrane–based formulations remains to be comprehensively evaluated (*53*, *54*). Developing safer biomimetic drug delivery systems with improved patient compliance will be the focus of our future investigations. In summary, regulating tumor cell life cycles holds broad therapeutic potential for precise ER delivery, antitumor immune activation, and tumor metastasis inhibition. The time engine nanosystem offers a promising perspective for the treatment of tumor metastasis.

## MATERIALS AND METHODS

### Materials and reagents

SA-β-Gal kit, rhodamine-conjugated phalloidin and matrigel were purchased from Yeasen Biotechnology Co. Ltd. (China). Propidium iodide (PI), Pal, Rib, and BFA were purchased from AbMole BioScience Inc. (USA). 3-(4,5-dimethylthiazol-2-yl)-2,5-diphenyltetrazolium bromide (MTT) was purchased from J&K Scientific (China). PLGA (50/50) was purchased from LACTEL (USA). Soybean phospholipid was purchased from Taiwei Pharmaceutical Co. (China). 1,2-Distearoyl-*sn*-glycero-3-phosphatidylethanolamine (DSPE)–PEG2000-Ome was purchased from Ponsure Biotechnology (China). Consumables related to WB and polyclonal antibodies (including BiP, ATF6, IRE1, HMGB1, ROCK1/2, vimentin, E-cadherin, VAMP2/4/8, caveolin-1, SNAP23, and iFluor 647 goat anti-rabbit/mouse IgG (H + L) were purchased from HUABIO Biotechnology Co. Ltd. (China). Penicillin-streptomycin (liquid), RPMI 1640 medium, FBS, and trypsin were purchased from Gibco (USA). Coumarin 6 (C6) was purchased from Sigma-Aldrich (USA). 1-Dioctadecyl-3,3,3,3-tetramethylindodicarbocyanine (DiR), 3,3′-dioctadecyloxacarbocyanine perchlorate (DiO), and 1,1′-dioctadecyl-3,3,3′,3′-tetramethylindocarbocyanine perchlorate (DiI) were purchased from Huayueyang Biotechnology Co. Ltd. (China). The ER extraction kit and crystal violet were purchased from Solarbio Science & Technology Co. Ltd. (China). The ER tracker was purchased from Beijing Labgic Technology Co. Ltd. (China). Enzyme-linked immunosorbent assay (ELISA) kits were purchased from Thermo Fisher Scientific Inc. (USA). Transwell was purchased from Millipore (USA). MHC-I antibody, CD80, CD86, CD3, CD4, CD8, FOXP3, CD11b, and Gr1 were purchased from BioLegend Inc. (USA). CRT antibody was purchased from Abcam plc (UK). Annexin V–fluorescein isothiocyanate (FITC) apoptosis detection kit was purchased from Elabscience Biotechnology Co. Ltd. (China). All other chemical reagents were of analytical grade.

### Cell lines and animals

Mouse breast cancer 4T1 cells were purchased from Icell Biotech Co. Ltd. (China). Cells were cultured in RPMI 1640 medium supplemented with 10% FBS and 1% penicillin-streptomycin, and maintained in a 5% CO_2_ atmosphere at 37°C. Female Balb/c mice (6 to 8 weeks old) were purchased from SPF Biotechnology Co. Ltd. (China). All animal experiments were approved by the Medical Ethics Committee of Sichuan University [approval number: SYXK (Chuan) 2023–0113].

### Screening for optimal concentration of senescence inducer

Cell cycle arrest and the expression of SA-β-Gal are typical characteristics of cellular senescence. Therefore, we screened the administration concentration of the senescence inducer through cell cycle assay and SA-β-Gal staining. For the cell cycle assay, 4T1 cells were treated with 1640++ medium, Rib (equivalent doses of 1, 5, and 10 μM), or Pal (equivalent doses of 1, 5, and 10 μM). After 24 hours of treatment, the cells were trypsinized, fixed with prechilled 70% ethanol overnight, and stained with PI at 5 μg/ml for 20 min. Cell cycle distribution was analyzed using flow cytometry, and the proportion of cells in each phase was quantified using FlowJo software. In a parallel manner, SA-β-Gal staining was performed using a commercial kit, and the number of blue-green SA-β-Gal–positive cells was counted under a microscope. To ensure that senescence induction occurred without significant cytotoxicity, we further evaluated the cytotoxicity of the two inducers. 4T1 cells were treated with 1640++ medium or varying concentrations of Rib and Pal (with the maximum concentration set at 160 μM) for 24 hours. Subsequently, MTT at a concentration of 500 μg/ml was added. Following an additional 4-hour incubation, dimethyl sulfoxide (DMSO) was added. The plates were shaken on a shaker for 15 min (37°C, 60 rpm), and the absorbance at 490 nm was measured using a microplate reader. The cell viability (%) was calculated using the following formula

Cell viability (%)=(AD−AB)/(AC−AB)×100%

where AD represents the absorbance of the drug-treated group, AC denotes the absorbance of the control group, and AB is the absorbance of the blank group.

### Observation of cellular morphological and cytoskeleton

4T1 cells were treated with 1640++ medium or Rib (at an equivalent dose of 10 μM). After 24 hours of treatment, cellular morphology was observed under an SEM to assess cell size and the number of cell pseudopodia. For cytoskeleton structure observation, 4T1 cells were treated with the same administration regimen as above. After 4 hours of treatment, the cells were fixed with 4% paraformaldehyde followed by permeabilization with 0.1% Triton X-100. Subsequently, the cells were stained with rhodamine-labeled phalloidin and then with 4′,6-diamidino-2-phenylindole (DAPI). Cytoskeletal morphology was observed using a confocal laser scanning microscope (CLSM).

### Extraction of normal and senescent cell membranes

4T1 cells were seeded separately into two cell culture dishes. For one dish, cells were cultured to approximately 90% confluence and then harvested as normal 4T1 cells. For the other dish, when cells reached around 60% confluence, they were induced to senescence with Rib (10 μM); after 24 hours of treatment, senescent 4T1 cells were collected. Cell pellets of both types were homogenized repeatedly and centrifuged (3000*g*, 10 min). The supernatant was collected, and all supernatants were pooled before a second centrifugation (10,000*g*, 10 min). The resulting supernatant was ultracentrifuged at 100,000*g* and 4°C for 1 hour. The obtained precipitate, corresponding to normal or senescent cell membranes (designated as N mem or S mem, respectively), was stored at −20°C for subsequent use.

### Protein profiles of normal and senescent 4T1 membranes

SDS loading buffer was added to N mem or S mem, and the samples were boiled for 10 min. The expression levels of proteins were analyzed via WB. Following the completion of electrophoresis, the gel was incubated with Coomassie Brilliant Blue overnight. Subsequent to washing with tris-buffered saline with Tween-20, images were captured to visualize the band distribution across all groups. In parallel, proteomic analysis was performed to identify and quantify differential proteins between the two membrane types. Bioinformatics analysis was subsequently conducted on the proteomic data, including volcano plot and heatmap analyses, to explore correlations between protein alterations and differential cellular/organellar targeting efficiencies.

### Preparation of cell membrane–coated nanoparticles

The PLGA nanoparticle core was prepared via the nanoprecipitation method. PLGA, soybean phosphatidylcholine (SPC), DSPE-PEG_2000_-Ome, and BFA were weighed at a mass ratio of 100:40:40:1 (w/w) and dissolved in 200 μl of DMSO. This solution was then slowly added dropwise into 4 ml of reverse osmosis (RO) water using a microfluidic device to form the PLGA core. Subsequently, normal or senescent cell membranes were added at a PLGA:membrane mass ratio of 2:1 (w/w). The mixture was subjected to probe sonication at 100 W for 5 min, followed by ultrafiltration concentration (100 kDa, 3000*g*, 10 min). The resulting product was washed three times with RO water to obtain membrane-coated nanoparticles (designated as B@NNP or B@SNP). For senescence inducer–loaded nanoparticles (R@SNP), PLGA, SPC, DSPE-PEG_2000_-Ome, and Rib were weighed at a mass ratio of 5:2:2:2 (w/w). The remaining steps were the same as above.

### Characterization of cell membrane–coated nanoparticles

The particle size, polydispersity index, and zeta potential of the nanoparticles were measured using a Malvern particle size analyzer. The drug encapsulation efficiency (EE%) was determined via high-performance liquid chromatography (HPLC). For the determination of BFA content, the mobile phase was composed of methanol-water (60:40, v/v) with a detection wavelength set at 230 nm. For Rib, the mobile phase was acetonitrile-water (containing 0.1% sodium heptanesulfonate and 2% triethylamine, with pH adjusted to 2.6 using phosphoric acid) (32:68, v/v), and the detection wavelength was 264 nm. The EE% and drug loading (DL%) were calculated using the following formulas: EE% = (*W*_NP_/*W*_D_) × 100%, DL% = (*W*_NP_/*W*_total_), where *W*_D_ refers to the total amount of drug added during preparation, *W*_NP_ denotes the amount of drug encapsulated in the nanoparticles, and *W*_total_ represents the total amount of the nanoparticles. The morphological characteristics of the nanoparticles were observed using a TEM. To assess storage stability, nanoparticles were dispersed in PBS (pH 7.4) and stored at 4°C, with particle size measured daily to evaluate their stability. In addition, nanoparticles were dispersed in 10% FBS (37°C, 60 rpm), and their stability in serum was assessed over 24 hours. For drug release assay, B@NNP, B@SNP, and R@SNP were separately placed into dialysis bags (*M*_W_: 8000 to 14,000 Da) and immersed in 50 ml of PBS (pH 7.4) under shaking conditions (37°C, 60 rpm). At predefined time points (0, 0.5, 1, 2, 4, 6, 8, 12, 24, 36, 48, 60 and 72 hours), 100 μl aliquots of the release medium were collected, replaced with an equal volume of fresh PBS, and analyzed by HPLC to determine drug concentrations. Cumulative drug release of Rib and BFA was calculated from peak areas detected at 264 and 230 nm, respectively.

### Verification of successful membrane coating on nanoparticle surfaces

SDS-PAGE was first used to confirm the successful coating of cell membranes on the nanoparticle surface. The test groups included senescent cell membranes (S mem), membrane-coated nanoparticles (B@SNP, R@SNP), and uncoated nanoparticles (B@NP, R@NP); the experimental procedures were consistent with those described above. To further evaluate the membrane coating efficiency, an FRET assay was additionally performed. Specifically, cell membranes were labeled with DiO, while DiI (another fluorescent dye, forming a FRET pair with DiO) was encapsulated in the core of PLGA nanoparticles. Subsequently, a microplate reader was used to detect the presence of the FRET effect—an observable FRET signal indicates effective membrane-nanoparticle binding, thereby verifying successful coating.

### Cellular uptake and internalization mechanisms of different nanoparticles

PLGA, SPC, DSPE-PEG_2000_-Ome, and C6 were weighed at a mass ratio of 200:80:80:1 (w/w), with the remaining preparation steps following those described above. To evaluate cellular uptake of the nanoparticles, 4T1 cells were treated with 1640++ medium (Control), NNP, or SNP (C6 = 1 μg/ml). After 4 hours of incubation, the cells were trypsinized, washed three times with PBS, and analyzed by flow cytometry to determine the fluorescence intensity. To further investigate the uptake mechanism, 4T1 cells were preincubated with uptake inhibitors for 2 hours. The remaining steps are consistent with those of the aforementioned cellular uptake experiment

### Evaluation of ER-targeting and retention ability of nanoparticles

To investigate the distribution of nanoparticles in the ER, 4T1 cells were treated with 1640++ medium, NNP, or SNP (C6 = 5 μg/ml). After 4 hours of incubation, the cells were harvested, and ER was isolated using an ER extraction kit. The fluorescence intensity of the isolated ER fractions was then measured using a microplate reader. For CLSM observation (to visualize nanoparticle-ER colocalization), 4T1 cells were treated with NNP or SNP (C6 = 5 μg/ml) for 4 hours. The cells were then stained with an ER tracker (for ER labeling) and DAPI (for nuclear staining), followed by observation under a CLSM.

### In vivo distribution study

DiR-loaded membrane-coated nanoparticles were prepared following the method described above. PLGA, SPC, DSPE-PEG_2000_-Ome, and DiR were weighed at a mass ratio of 200:80:80:1 (w/w) and processed following the same protocol. 4T1 cells in the logarithmic growth phase were trypsinized, centrifuged, and resuspended in PBS. A total of 4 × 10^5^ cells per mouse were inoculated into the third mammary fat pad of BALB/c mice to establish tumor models. When the tumor volume reached approximately 400 mm^3^, NNP or SNP was administered via tail vein injection at a DiR equivalent dose of 62.5 μg/kg. At predefined time points (2, 6, 12, 24, and 48 hours), the accumulation of nanoparticles in tumor tissues was visualized using an in vivo imaging system. After 48 hours, the mice were euthanized, and the major organs (heart, liver, spleen, lung and kidney) and tumors were collected for ex vivo fluorescence intensity analysis and semiquantitative assessment.

### Determination of the stiffness of cell membrane–coated nanoparticles

Nanoparticle suspension (0.05 ml) was dispensed onto the surface of freshly cleaved mica. The sample was then air-dried at room temperature and 85% relative humidity for 1 hour. Subsequently, an atomic force microscope (Bruker) was used for scanning, and the Young’s modulus of the nanoparticles was calculated from the acquired scanning data.

### Quantification MHC-I and SASP in tumor cells

To assess MHC-I expression and SASP in tumor cells, 4T1 cells were treated with 1640++ medium (N), NNP, or SNP for 24 hours. For MHC-I content determination, cells were incubated with MHC-I primary antibody at 4°C for 2 hours. The level of MHC-I on the cell surface was then measured by flow cytometry. In parallel, cell culture supernatants were collected to quantify SASP factors (including IFN-γ, TNF-α, and IL-1β) using ELISA kits.

### Mechanism of senescent membranes elevating tumor MHC-I

Senescent 4T1 cells were treated with 1640++ medium (Control), SNP, or SNP that had been preincubated with anti–MHC-I. After the 24-hour treatment, the Control and SNP groups were further incubated with anti–MHC-I. All three groups were then analyzed by flow cytometry to compare MHC-I–related differences. To further track the intracellular movement trajectory of MHC-I molecules derived from senescent tumor cell membranes, senescent 4T1 cells were treated with SNP preincubated with MHC-I antibody. The distribution of MHC-I at different time points was captured using a CLSM to visualize its dynamic movement.

### Senescent tumor cell membranes activate immunity within tumor model

The method for establishing the breast cancer models was consistent with that described above. When the tumor volume reached 100 mm^3^, the mice were injected intravenously with saline (N), B@NNP, or B@SNP (BFA = 0.3 mg/kg) every 3 days for a total of five administrations. When tumors in the N group reached approximately 1500 mm^3^, the mice were euthanized. Tumors were weighed, lung tissues were fixed, and lung nodules were counted. After grinding lymph nodes and tumor tissues, the infiltration of mature dendritic cells was determined, and the contents of MHC-I and SASP components in tumor cells were examined.

### Tumor distribution profiles of two drugs following sequential administration

The method for establishing the breast cancer models was consistent with that described above. When the tumor volume reached 500 mm^3^, the mice were injected intravenously with R@SNP (Rib = 4.5 mg/kg), followed by administration of B@SNP (BFA = 3 mg/kg) 24 hours later. At predetermined time points, tumor tissues were harvested and repeatedly homogenized with methanol-acetonitrile mixture to extract analytes. The organic phase was dried under a nitrogen stream, and the residue was reconstituted in methanol before HPLC analysis. The HPLC detection conditions were identical to those outlined above.

### Efficacy of the sequential strategy against tumor metastasis in vitro

To evaluate the cytotoxicity of different preparations, 4T1 cells were treated with 1640++ medium (N), ER-targeting nanoparticles (B@SNP, BFA = 1 μM), senescence inducer (R@SNP, Rib = 15 μM) for 24 hours, or sequential administration [Seqtl., R@SNP (Rib = 15 μM) was administered first for 24 hours and then B@SNP (BFA = 1 μM) for another 24 hours]. The interval for the subsequent in vitro sequential treatment regimen was consistent with the protocol described above. The remaining steps were performed as described above. For the migration assay, 4T1 cells were seeded in the upper chambers of Transwell, with serum-containing medium added to the lower chambers. After incubation for 4 hours, the cells were treated with N, B@SNP (BFA = 1 μM), R@SNP (Rib = 15 μM), and Seqtl. (Rib = 15 μM → BFA = 1 μM). Following 24 hours of incubation, the cells were fixed with 4% paraformaldehyde, permeabilized with 0.1% Triton X-100, and then stained with crystal violet (1 mg/ml) for 10 min, respectively. Migrated cells in the lower chamber were observed under a microscope. Subsequently, crystal violet was solubilized with 33% glacial acetic acid (v/v), and the absorbance at 570 nm was measured using a microplate reader. The relative migration rate was calculated as follows: Migration rate = (*A*_S_ − *A*_0_) / (*A*_C_ − *A*_0_) × 100%, where *A*_S_ represents the absorbance of various treated groups, *A*_0_ denotes the negative control group, and *A*_C_ is the 1640++ medium–treated group. For invasion assay, matrigel was diluted to 3 mg/ml with serum-free medium and added to the upper chamber of Transwell, followed by incubation in a cell culture incubator for 1 hour to allow solidification. The subsequent steps were identical to the migration assay.

### Mechanism of the sequential strategy against tumor metastasis in vitro

4T1 cells were treated with N, B@SNP (BFA = 1 μM), R@SNP (Rib = 15 μM), or Seqtl. (Rib = 15 μM → BFA = 1 μM) for 24 hours. After digestion, the cells were incubated with BiP, ATF6, and IRE1 antibodies, respectively, and the expression levels of these proteins were detected by flow cytometry. The expression of ROCK1/ROCK2 proteins (which mediate cytoskeleton remodeling) was examined via WB. To further investigate ER alterations, 4T1 cells were treated following the same protocol as described above. ER morphology of cells in different groups was observed using TEM.

### Efficacy of the sequential strategy against tumor metastasis in vivo

The mouse orthotopic breast cancer model was established following the method described above. When tumor volumes reached approximately 100 mm^3^, the mice were administered via tail vein injection with the following groups: saline-treated group (N), B@SNP-treated group [B@SNP, BFA (0.3 mg/kg)], R@SNP-treated group [R@SNP, Rib (4.5 mg/kg)], the concurrent administration group [Simul., BFA (0.3 mg/kg) + Rib (4.5 mg/kg)], and the sequential administration group [Seqtl., R@SNP (Rib = 4.5 mg/kg) was administered first, followed by B@SNP (BFA = 0.3 mg/kg) 24 hours later]. The interval for the subsequent in vivo sequential treatment regimen was consistent with the protocol described above. Administration was performed once every 3 days for a total of five doses. Mouse body weight and tumor volume were recorded every 2 days. When tumors in the saline group reached approximately 1500 mm^3^, the mice were euthanized. Subsequently, tumors and lungs were harvested to record tumor weight and the number of lung nodules, thereby assessing the antitumor growth and antimetastasis efficacy of the formulations. Immune cells, such as mature dendritic cells, CD8^+^ T cells, T_reg_ cells, and MDSC cells, were labeled with different markers and then detected for their infiltration via flow cytometry.

### Mechanisms of the sequential strategy against tumor metastasis in vivo

Tumor tissues from different administration groups in the aforementioned in vivo efficacy experiment were homogenized to prepare single-cell suspensions, which were then incubated with antibodies against BiP, ATF6, IRE1, CRT, and ROCK1/2, respectively. The expression levels of these proteins were detected by flow cytometry. The supernatant from homogenized tumor tissues was used to measure HMGB1 content via an ELISA kit, to determine the level of HMGB1 released by tumor cells. In addition, the levels of immunity-related cytokines (IFN-γ, IL-1β, and TNF-α) and metastasis-related cytokines (IL-6 and IL-10) in tumor tissues were detected using ELISA kits.

### Safety assessment of the sequential strategy in vivo

Whole blood was collected from mice in different administration groups of the above in vivo efficacy experiment. Blood routine analysis and blood biochemistry tests were then performed to evaluate the in vivo safety of the formulations. Moreover, liver, spleen, and tumor tissues (the top three with the highest nanoparticle accumulation in the in vivo distribution experiment) were harvested from the saline and Seqtl. groups. These tissues were stained using a SA-β-gal staining kit, and the number of senescent cells (pointed out by red arrows) was observed.

### Statistical analysis

Statistical analyses were performed using SPSS 19.0 software (SPSS, Chicago, IL, USA). All data were expressed as means ± SD. Student’s *t* test was applied for statistical analysis between two independent groups. One-way analysis of variance (ANOVA) was used to determine significant differences among multiple groups, followed by Tukey’s multiple comparisons. A *P* value <0.05 was considered statistically significant.

## References

[1] Z. Li, M. Li, J. Lu, Biomimetic liposomes in drug delivery: From design mechanisms to applications. Chem. Soc. Rev. 55, 1008–1038 (2026). 10.1039/d5cs00440c PMC12688027 41364452

[2] H. Xiao, F. Raza, K. Li, J. Song, H. Zafar, S. Yang, J. Su, M. Qiu, Cell membrane derived biomimetic nanomedicine for precision delivery of traditional Chinese medicine in cancer therapy. J. Control. Release 383, 113829 (2025). 10.1016/j.jconrel.2025.113829 40355044

[3] T. Liu, Q. Lu, H. Zhang, F. Dong, Z. He, J. Sun, B. Sun, Biomimetic drug delivery for anticancer: Focusing on the relationship between drugs and biomimetic carriers. Coord. Chem. Rev. 524, 216328 (2025).

[4] H. Sabit, T. M. Pawlik, F. Radwan, M. Abdel-Hakeem, S. Abdel-Ghany, A.-H. S. Wadan, M. Elzawahri, A. El-Hashash, B. Arneth, Precision nanomedicine: Navigating the tumor microenvironment for enhanced cancer immunotherapy and targeted drug delivery. Mol. Cancer 24, 160 (2025). 10.1186/s12943-025-02357-z PMC12131435 40457437

[5] V. Rahamim, A. Azagury, Bioengineered biomimetic and bioinspired noninvasive drug delivery systems. Adv. Funct. Mater. 31, 2102033 (2021).

[6] X. Zhang, G. Chen, H. Zhang, L. Shang, Y. Zhao, Bioinspired oral delivery devices. Nat. Rev. Bioeng. 1, 208–225 (2023).

[7] H. Liu, M. Huang, J. Lyu, J. Tao, Y. Li, L. Li, Y. Huang, Z. Zhou, Enhance efferocytosis and block the macrophages–platelets feedback loop for targeted treatment of psoriasis. ACS Nano 19, 11774–11791 (2025). 10.1021/acsnano.4c13533 40113447

[8] L. Zhu, X. Yu, T. Cao, H. Deng, X. Tang, Q. Lin, Q. Zhou, Immune cell membrane-based biomimetic nanomedicine for treating cancer metastasis. Acta Pharm. Sin. B 13, 2464–2482 (2023). 10.1016/j.apsb.2023.03.004 PMC10326251 37425034

[9] J. A. Finbloom, C. Huynh, X. Huang, T. A. Desai, Bioinspired nanotopographical design of drug delivery systems. Nat. Rev. Bioeng. 1, 139–152 (2023).

[10] M. Huang, H. Liu, J. Wu, J. Tao, Y. Wang, L. Li, Y. Huang, Z. Zhou, Engineered nanosponge platform corrects TLR7-related efferocytosis and platelet-activation for treatment of different systemic lupus erythematosus stages. Adv. Funct. Mater. 36, e14092 (2025).

[11] J. Lee, W. Sim, H. Park, B. Park, Y. K. Joung, Targeted delivery of apoptotic cell-derived nanovesicles prevents cardiac remodeling and attenuates cardiac function exacerbation. Adv. Funct. Mater. 33, 2210864 (2023).

[12] Y. You, J. Xu, Y. Liu, H. Li, L. Xie, C. Ma, Y. Sun, S. Tong, K. Liang, S. Zhou, F. Ma, Q. Song, W. Xiao, K. Fu, C. Dai, S. Li, J. Lei, Q. Mei, X. Gao, J. Chen, Tailored apoptotic vesicle delivery platform for inflammatory regulation and tissue repair to ameliorate ischemic stroke. ACS Nano 17, 8646–8662 (2023). 10.1021/acsnano.3c01497 37099675

[13] J. Shi, W. Wu, D. Chen, Z. Liao, T. Sheng, Y. Wang, Y. Yao, Q. Wu, F. Liu, R. Zhou, C. Zhu, X. Shen, Z. Mao, Y. Ding, W. Wang, G. Dotti, J. Sun, X. Liang, W. Fang, P. Zhao, H. Li, Z. Gu, Lyophilized lymph nodes for improved delivery of chimeric antigen receptor T cells. Nat. Mater. 23, 844–853 (2024). 10.1038/s41563-024-01825-z 38448658

[14] S. Dalton, Linking the cell cycle to cell fate decisions. Trends Cell Biol. 25, 592–600 (2015). 10.1016/j.tcb.2015.07.007 PMC4584407 26410405

[15] Y. Wang, R. Li, R. Tong, T. Chen, M. Sun, L. Luo, Z. Li, Y. Chen, Y. Zhao, C. Zhang, L. Wei, W. Lin, H. Chen, K. Qian, A. F. Chen, J. Liu, L. Chen, B. Li, F. Wang, L. Wang, B. Su, J. Pu, Integrating single-cell RNA and T cell/B cell receptor sequencing with mass cytometry reveals dynamic trajectories of human peripheral immune cells from birth to old age. Nat. Immunol. 26, 308–322 (2025). 10.1038/s41590-024-02059-6 PMC11785523 39881000

[16] J. P. De Magalhães, Cellular senescence in normal physiology. Science 384, 1300–1301 (2024). 10.1126/science.adj7050 38900869

[17] S. Jaillon, A. Ponzetta, D. Di Mitri, A. Santoni, R. Bonecchi, A. Mantovani, Neutrophil diversity and plasticity in tumour progression and therapy. Nat. Rev. Cancer 20, 485–503 (2020). 10.1038/s41568-020-0281-y 32694624

[18] P. Foucambert, J. W. Hang, B. Malleret, Exploring erythropoiesis: Aging through the lens of malaria and microgravity. Trends Parasitol. 41, 1046–1061 (2025). 10.1016/j.pt.2025.09.008 41093725

[19] L. Chen, C. Liu, Y. Xiang, J. Lyu, Z. Zhou, T. Gong, H. Gao, L. Li, Y. Huang, Exocytosis blockade of endoplasmic reticulum-targeted nanoparticle enhances immunotherapy. Nano Today 42, 101356 (2022).

[20] L. Chen, P. Jiang, X. Shen, J. Lyu, C. Liu, L. Li, Y. Huang, Cascade delivery to Golgi apparatus and on-site formation of subcellular drug reservoir for cancer metastasis suppression. Small 19, e2204747 (2023). 10.1002/smll.202204747 36585358

[21] Y. Deng, F. Jia, P. Jiang, L. Chen, L. Xing, X. Shen, L. Li, Y. Huang, Biomimetic nanoparticle synchronizing pyroptosis induction and mitophagy inhibition for anti-tumor therapy. Biomaterials 301, 122293 (2023). 10.1016/j.biomaterials.2023.122293 37639978

[22] M. Colucci, M. Sarill, M. Maddalena, A. Valdata, M. Troiani, M. Massarotti, M. Bolis, S. Bressan, A. Kohl, D. Robesti, M. Saponaro, Q. Shi, P. Song, D. Brina, B. Calì, A. Alimonti, Senescence in cancer. Cancer Cell 43, 1204–1226 (2025). 10.1016/j.ccell.2025.05.015 40513577

[23] H. Lee, S. Lee, Y. Seo, D. Kim, Y. Oh, J. Jin, B. Hyeon, Y. Han, H. Kim, Y. J. Lee, H. M. Kim, G. Lee, K.-H. Cho, W. Do Heo, A Rho GTPase-effector ensemble governs cell migration behavior. Nat. Commun. 16, 9637 (2025). 10.1038/s41467-025-64635-0 PMC12579213 41173885

[24] Z. Lin, X. Chen, X.-Q. Feng, Adaptive cytoskeletal responses to extracellular environment viscosity modulate cell migration. ACS Nano 20, 275–287 (2026). 10.1021/acsnano.5c11355 41469235

[25] Y. Hui, X. Yi, D. Wibowo, G. Yang, A. P. J. Middelberg, H. Gao, C.-X. Zhao, Nanoparticle elasticity regulates phagocytosis and cancer cell uptake. Sci. Adv. 6, eaaz4316 (2020). 10.1126/sciadv.aaz4316 PMC7164958 32426455

[26] A. C. Dumitru, D. Mohammed, M. Maja, J. Yang, S. Verstraeten, A. Del Campo, M. Mingeot-Leclercq, D. Tyteca, D. Alsteens, Label-free imaging of cholesterol assemblies reveals hidden nanomechanics of breast cancer cells. Adv. Sci. 7, 2002643 (2020). 10.1002/advs.202002643 PMC7675049 33240781

[27] J. Gao, F. Yang, Y. Jiang, Y. Zheng, L. Cai, X. Huang, L. Yuan, Y. Wang, Y. Wang, Z. Xu, TAPT1 interacts with SUCO to maintain the homeostasis of newly synthesized proteins and brain development in mice. Proc. Natl. Acad. Sci. U.S.A. 122, e2501361122 (2025). 10.1073/pnas.2501361122 PMC12718307 41379998

[28] I. Marin, O. Boix, A. Garcia-Garijo, I. Sirois, A. Caballe, E. Zarzuela, I. Ruano, C. S.-O. Attolini, N. Prats, J. A. López-Domínguez, M. Kovatcheva, E. Garralda, J. Muñoz, E. Caron, M. Abad, A. Gros, F. Pietrocola, M. Serrano, Cellular senescence is immunogenic and promotes antitumor immunity. Cancer Discov. 13, 410–431 (2023). 10.1158/2159-8290.CD-22-0523 PMC7614152 36302218

[29] T. N. M. Pham, N. Perumal, C. Manicam, M. Basoglu, S. Eimer, D. C. Fuhrmann, C. U. Pietrzik, A. M. Clement, H. Körschgen, J. Schepers, C. Behl, Adaptive responses of neuronal cells to chronic endoplasmic reticulum (ER) stress. Redox Biol. 67, 102943 (2023). 10.1016/j.redox.2023.102943 PMC10618786 37883843

[30] J. Moral-Sanz, I. Fernández-Carrasco, V. Ramponi, A. Garrido, I. Karbat, P. Cabezas-Sainz, E. Rivera-de-Torre, O. Elsallabi, R. Martín-Hernández, J. L. López-Aceituno, N. L. Price, L. Sanchez, G. Colmenarejo, Á. Martínez-del-Pozo, I. Vetter, A. Cogolludo, F. Perez-Vizcaino, J. Del-Pozo, E. Reuveny, M. A. Fernández-Rojo, P. D. Robbins, R. De Cabo, M. Serrano, M. P. Ikonomopoulou, Senotoxins target senescence via lipid binding specificity, ion imbalance and lipidome remodeling. Nat. Aging 6, 349–367 (2026). 10.1038/s43587-025-01030-w PMC12920127 41526742

[31] Z. Dong, Y. Luo, Z. Yuan, Y. Tian, T. Jin, F. Xu, Cellular senescence and SASP in tumor progression and therapeutic opportunities. Mol. Cancer 23, 181 (2024). 10.1186/s12943-024-02096-7 PMC11365203 39217404

[32] X. Chen, C. Shi, M. He, S. Xiong, X. Xia, Endoplasmic reticulum stress: Molecular mechanism and therapeutic targets. Signal Transduct. Target. Ther. 8, 352 (2023). 10.1038/s41392-023-01570-w PMC10502142 37709773

[33] A. Fu, B. Yao, T. Dong, Y. Chen, J. Yao, Y. Liu, H. Li, H. Bai, X. Liu, Y. Zhang, C. Wang, Y. Guo, N. Li, S. Cai, Tumor-resident intracellular microbiota promotes metastatic colonization in breast cancer. Cell 185, 1356–1372.e26 (2022). 10.1016/j.cell.2022.02.027 35395179

[34] J. Zhao, A. Tong, J. Liu, M. Xu, P. Mi, Tumor-targeting nanocarriers amplified immunotherapy of cold tumors by STING activation and inhibiting immune evasion. Sci. Adv. 11, eadr1728 (2025). 10.1126/sciadv.adr1728 PMC12204131 40577452

[35] C. T. Kureshi, S. K. Dougan, Cytokines in cancer. Cancer Cell 43, 15–35 (2025). 10.1016/j.ccell.2024.11.011 PMC11841838 39672170

[36] C.-H. Hung, S.-Y. Wu, C.-I. D. Yao, H.-H. Yeh, C.-C. Lin, C.-Y. Chu, T.-Y. Huang, M.-R. Shen, C.-H. Lin, W.-C. Su, Defective N-glycosylation of IL6 induces metastasis and tyrosine kinase inhibitor resistance in lung cancer. Nat. Commun. 15, 7885 (2024). 10.1038/s41467-024-51831-7 PMC11385228 39251588

[37] A. M. Shiri, T. Zhang, T. Bedke, D. E. Zazara, L. Zhao, J. Lücke, M. Sabihi, A. Fazio, S. Zhang, D. V. F. Tauriello, E. Batlle, B. Steglich, J. Kempski, T. Agalioti, M. Nawrocki, Y. Xu, K. Riecken, I. Liebold, L. Brockmann, L. Konczalla, L. Bosurgi, B. Mercanoglu, P. Seeger, N. Küsters, P. M. Lykoudis, A. Heumann, P. C. Arck, B. Fehse, P. Busch, R. Grotelüschen, O. Mann, J. R. Izbicki, T. Hackert, R. A. Flavell, N. Gagliani, A. D. Giannou, S. Huber, IL-10 dampens antitumor immunity and promotes liver metastasis via PD-L1 induction. J. Hepatol. 80, 634–644 (2024). 10.1016/j.jhep.2023.12.015 PMC10964083 38160941

[38] Y. Sun, J.-P. Coppé, E. W.-F. Lam, Cellular senescence: The sought or the unwanted? Trends Mol. Med. 24, 871–885 (2018). 10.1016/j.molmed.2018.08.002 30153969

[39] T. Yoneda, M. A. Alsina, J. B. Chavez, L. Bonewald, R. Nishimura, G. R. Mundy, Evidence that tumor necrosis factor plays a pathogenetic role in the paraneoplastic syndromes of cachexia, hypercalcemia, and leukocytosis in a human tumor in nude mice. J. Clin. Invest. 87, 977–985 (1991). 10.1172/JCI115106 PMC329890 1999505

[40] S. Chen, Y. Li, Z. Zhou, Q. Saiding, Y. Zhang, S. An, M. M. Khan, X. Ji, R. Qiao, W. Tao, N. Kong, W. Chen, T. Xie, Macrophage hitchhiking nanomedicine for enhanced β-elemene delivery and tumor therapy. Sci. Adv. 11, eadw7191 (2025). 10.1126/sciadv.adw7191 PMC12094207 40397726

[41] M. Zhou, Y. Tang, Y. Lu, T. Zhang, S. Zhang, X. Cai, Y. Lin, Framework nucleic acid-based and neutrophil-based nanoplatform loading baicalin with targeted drug delivery for anti-inflammation treatment. ACS Nano 19, 3455–3469 (2025). 10.1021/acsnano.4c12917 39817852

[42] B. Xie, H. Zhao, Y. Ding, Z. Wang, C. Gao, S. Li, K. Zhang, S. W. Ip, H. Yu, R. Wang, Supramolecularly engineered conjugate of bacteria and cell membrane-coated magnetic nanoparticles for enhanced ferroptosis and immunotherapy of tumors. Adv. Sci. 10, e2304407 (2023). 10.1002/advs.202304407 PMC10700203 37850572

[43] N. Wen, Y. Lu, Y. Zhuo, B. Fu, H. Wang, Y. He, H. Wu, Z. Wang, W. Tan, L. Qiu, Enhancing T-cell infiltration and immunity in solid tumors via DNA nanolinker-mediated monocyte hitchhiking. J. Am. Chem. Soc. 147, 9800–9809 (2025). 10.1021/jacs.4c18455 40042588

[44] Y. He, C. Cheng, Y. Liu, F.-M. Chen, Y. Chen, C. Yang, Z. Zhao, J. Dawulieti, Z. Shen, Y. Zhang, J.-Z. Du, S. Guan, D. Shao, Intravenous senescent erythrocyte vaccination modulates adaptive immunity and splenic complement production. ACS Nano 18, 470–482 (2024). 10.1021/acsnano.3c07943 38146673

[45] Z. Luo, Y. Lu, Y. Shi, M. Jiang, X. Shan, X. Li, J. Zhang, B. Qin, X. Liu, X. Guo, J. Huang, Y. Liu, S. Wang, Q. Li, L. Luo, J. You, Neutrophil hitchhiking for drug delivery to the bone marrow. Nat. Nanotechnol. 18, 647–656 (2023). 10.1038/s41565-023-01374-7 37081080

[46] F. Liu, S. Ning, X. Wang, X. Fan, B. Wan, F. Sun, L. Du, K. Shi, X. Zou, R. Zhu, M. Li, W. Shen, Z. He, K. Wang, J. Sun, Pulsatile sequential drug release system for cascade tumor deep penetration and differentiation therapy to enhance chemoimmunotherapy. Sci. Adv. 11, eadr8001 (2025). 10.1126/sciadv.adr8001 PMC12407085 40901943

[47] Y. Peng, W. Feng, H. Huang, Y. Chen, S. Yang, Macrophage-targeting antisenescence nanomedicine enables in-situ NO induction for gaseous and antioxidative atherosclerosis intervention. Bioact. Mater. 48, 294–312 (2025). 10.1016/j.bioactmat.2025.02.025 PMC11889351 40060144

[48] L. Li, M. Zhang, J. Li, T. Liu, Q. Bao, X. Li, J. Long, L. Fu, Z. Zhang, S. Huang, Z. Liu, L. Zhang, Cholesterol removal improves performance of a model biomimetic system to co-deliver a photothermal agent and a STING agonist for cancer immunotherapy. Nat. Commun. 14, 5111 (2023). 10.1038/s41467-023-40814-9 PMC10444796 37607938

[49] Y. Cheng, Z. Qu, Q. Jiang, T. Xu, H. Zheng, P. Ye, M. He, Y. Tong, Y. Ma, A. Bao, Functional materials for subcellular targeting strategies in cancer therapy: Progress and prospects. Adv. Mater. 37, e2305095 (2025). 10.1002/adma.202305095 37665594

[50] J. Soukar, N. A. Peppas, A. K. Gaharwar, Organelle-targeting nanoparticles. Adv. Sci. 12, e2411720 (2025). 10.1002/advs.202411720 PMC11831507 39806939

[51] H. Yang, D. Liu, L. Qiu, R. Wang, C. Zhang, D. Yu, Q. Zhong, N. Yuki, Z. Song, T. Zhu, H. Ju, W. Hong, J. Zhu, Reprogramming cellular senescence and aging clocks for advanced cancer immunotherapy. Mol. Cancer 24, 237 (2025). 10.1186/s12943-025-02459-8 PMC12492661 41039629

[52] X. Shen, Y. Deng, L. Chen, C. Liu, L. Li, Y. Huang, Modulation of autophagy direction to enhance antitumor effect of endoplasmic-reticulum-targeted therapy: Left or right? Adv. Sci. 10, e2301434 (2023). 10.1002/advs.202301434 PMC10427372 37290058

[53] J.-J. Zhao, S.-N. Tian, Z.-Y. Peng, J.-X. Ren, Y.-Y. Zhang, G.-H. Li, D.-H. Deng, J.-P. Zhang, X.-B. Zhang, Biomembrane-inspired lipid nanoparticles enhance CRISPR-Cas9 editing for hemophilia A. J. Control. Release 386, 114141 (2025). 10.1016/j.jconrel.2025.114141 40834922

[54] Y. An, C. Ji, H. Zhang, Q. Jiang, M. F. Maitz, J. Pan, R. Luo, Y. Wang, Engineered cell membrane coating technologies for biomedical applications: From nanoscale to macroscale. ACS Nano 19, 11517–11546 (2025). 10.1021/acsnano.4c16280 40126356

