# Supplementary material for: Senescent tumor cell membrane–based “time engine” nanosystem amplifies endoplasmic reticulum targeting for metastasis blockade
Source: Sci Adv. 2026 Oct 9;12(41):eaeh0494. doi: 10.1126/sciadv.aeh0494 (PMC13651867; doi:10.1126/sciadv.aeh0494)
Supplement: Supplementary file 1 [file sciadv.aeh0494_sm.pdf]

Science Advances
AAAS

Supplementary Materials for

Senescent tumor cell membrane–based "time engine" nanosystem amplifies endoplasmic reticulum targeting for metastasis blockade

Jing Tao *et al.*

Corresponding author: Yuan Huang, huangyuan0@163.com

Sci. Adv. **12**, eaeh0494 (2026)
DOI: 10.1126/sciadv.aeh0494

This PDF file includes:

Figs. S1 to S16
Table S1

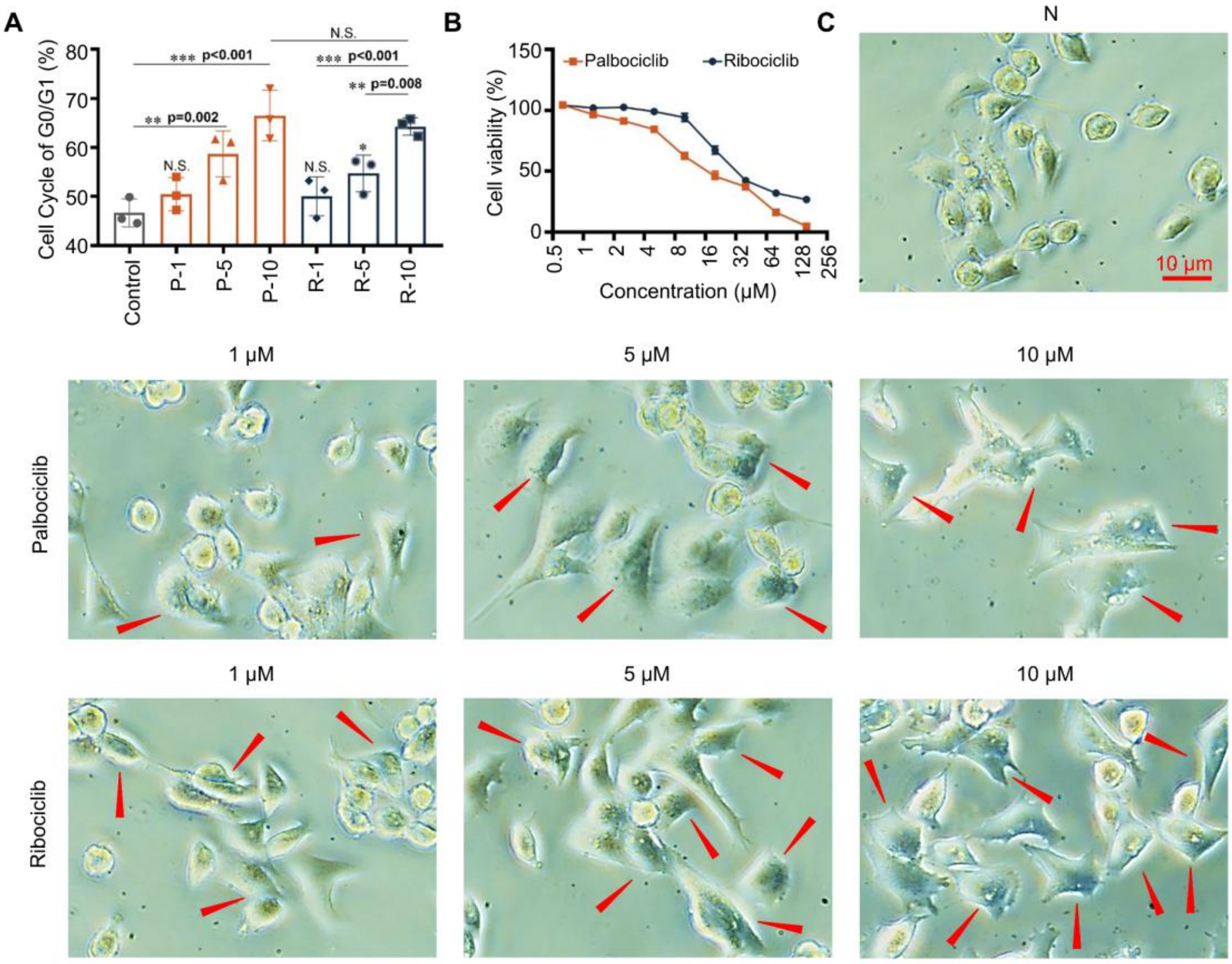

Fig. S1. Screening of senescence inducers. Cell cycle arrest (A), Cell viability (B), and detection of senescence-associated β-galactosidase (SA-β-gal) expression (C) at various concentrations of palbociclib (Pal) and ribociclib (Rib). Scale bar, 10 μm. The red arrow indicates senescent cells. Data are presented as mean ± standard deviation (SD), with n = 3. Statistical significance ($p<0.05$) was determined by one-way ANOVA test.

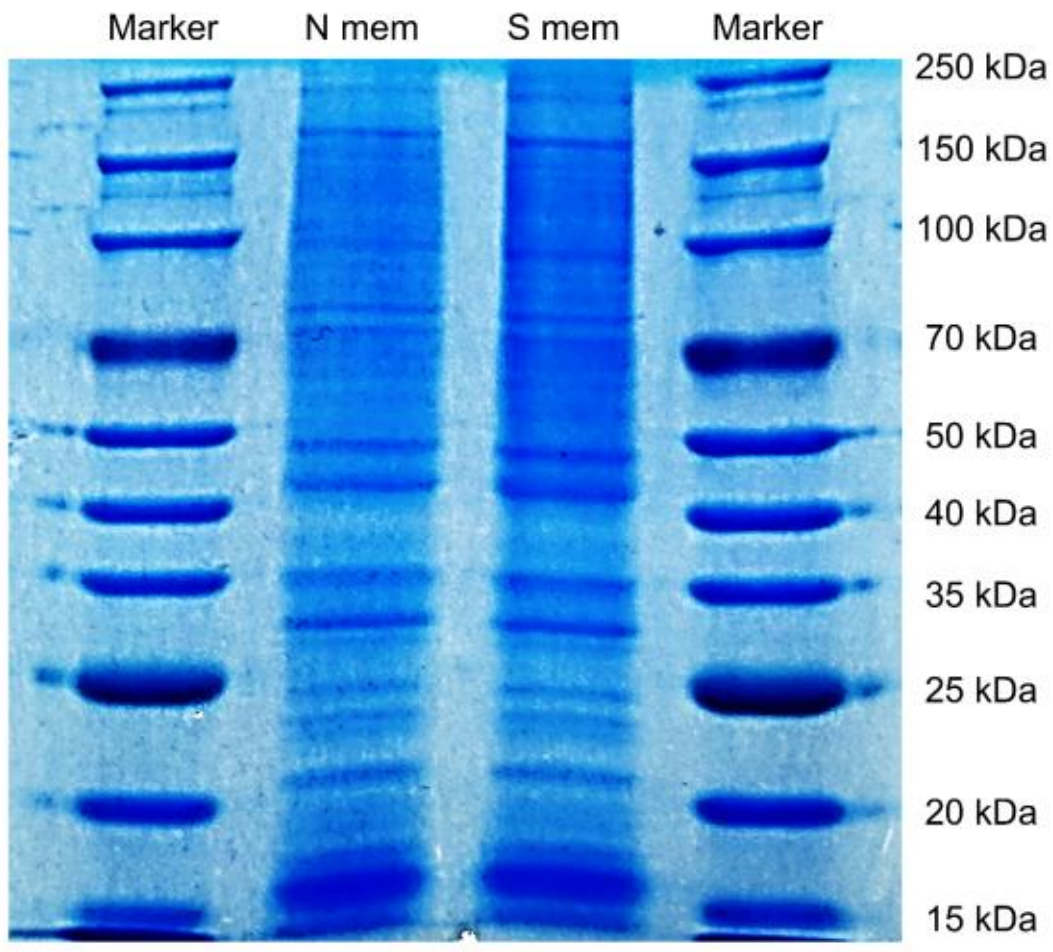

Fig. S2. SDS-PAGE analysis of membrane protein profile disparities between normal (N) and senescent (S) 4T1 cells.

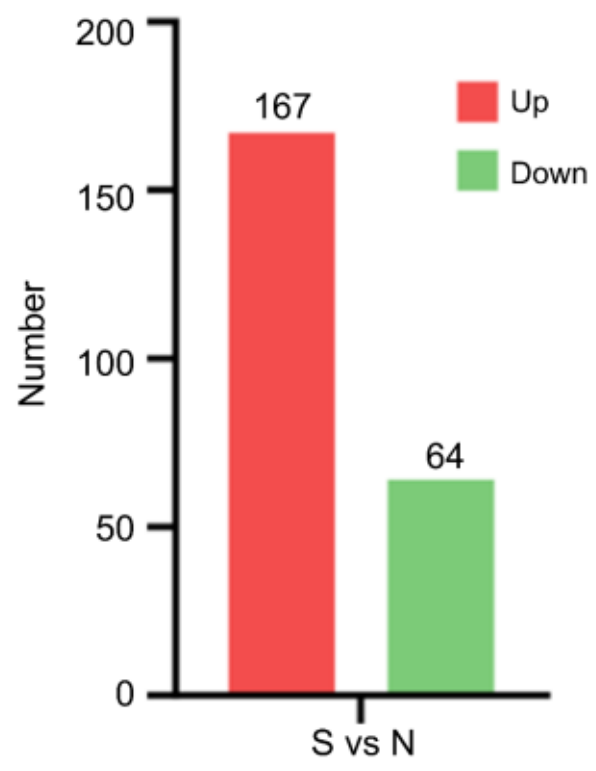

Fig. S3. Proteomics analysis of membrane protein profile disparities between normal (N) and senescent (S) 4T1 cells.

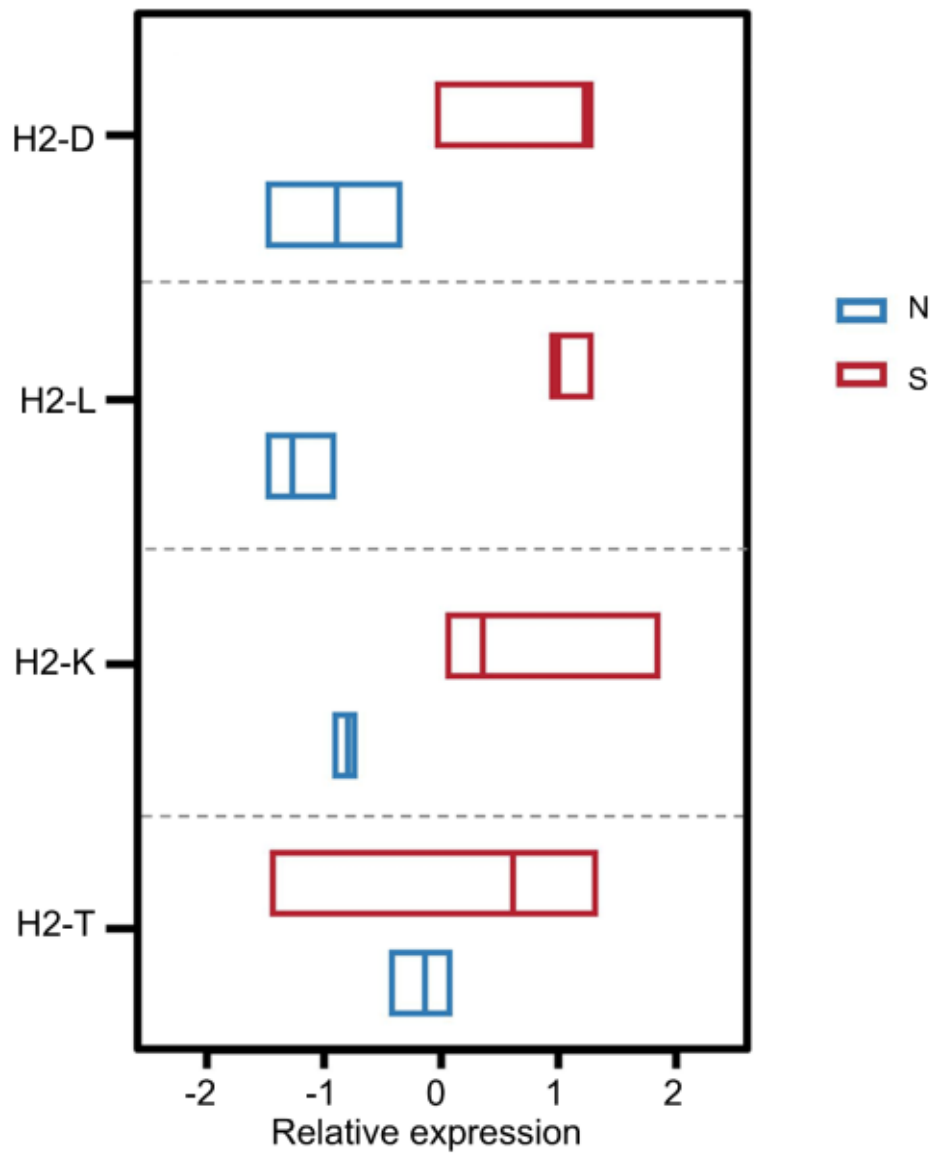

Fig. S4. Proteomics analysis of major histocompatibility complex class I (MHC-I) molecules.

	**Size (nm)**	**PDI**	**Zeta potential (mV)**	**EE (%)**	**DL (%)**
N mem	146.1±1.0	0.160±0.014	-19.5±0.8	-	-
S mem	152.1±1.0	0.134±0.041	-26.7±0.6	-	-
B@NP	92.8±1.0	0.172±0.040	-23.4±0.7	68.6±1.1	0.38±0.06
B@NNP	136.7±0.3	0.196±0.000	-28.3±1.2	69.7±0.9	0.34±0.03
B@SNP	146.0±2.8	0.137±0.028	-36.3±0.4	72.1±1.6	0.37±0.04
R@NP	52.5±0.4	0.242±0.010	-14.7±0.4	43.0±0.7	6.13±0.64
R@SNP	126.5±1.4	0.259±0.012	-30.1±1.5	42.8±2.3	6.04±0.99

Table S1. Characterization of nanoparticles.

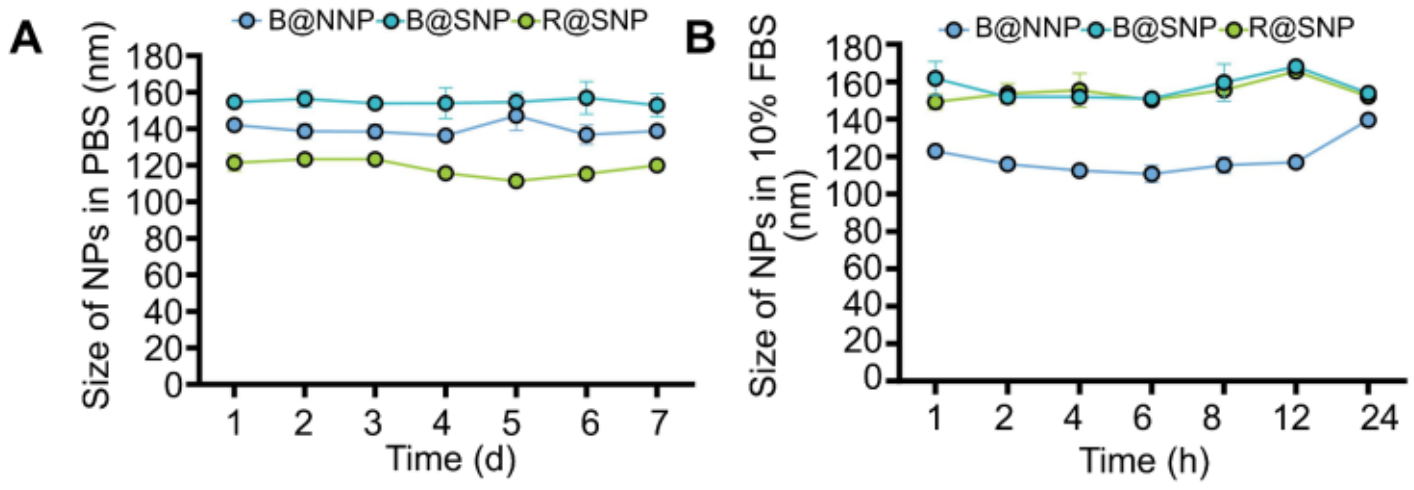

Fig. S5. Stability investigation of nanoparticles. (A) In PBS (pH 7.4). (B) In 10% fetal bovine serum. Data are presented as mean ± standard deviation (SD), with n = 3.

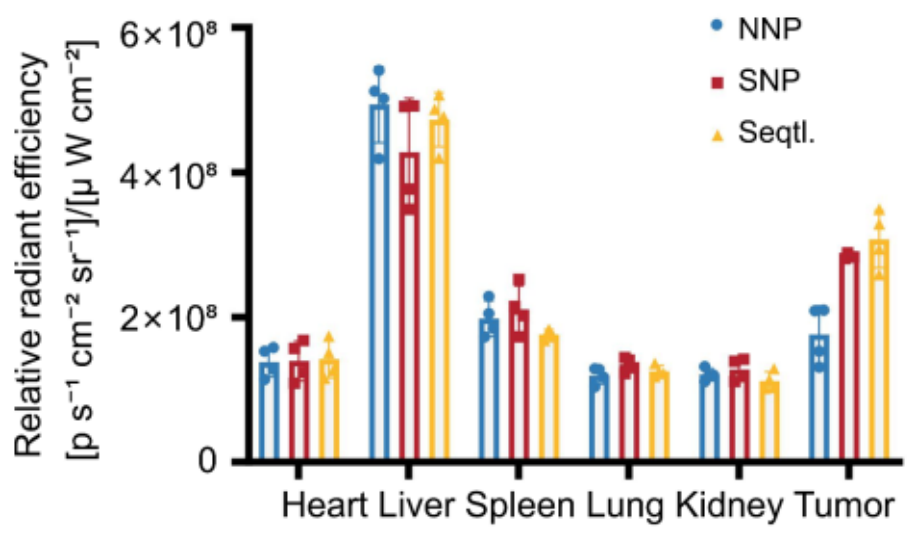

Fig. S6. Accumulation of membrane-coated nanoparticles in mouse organs at 48 h after intravenous tail-vein nanoparticle administration. Group Seqtl. represented treatment with R@SNP, followed by B@SNP administration 24 h later. Data are presented as mean ± standard deviation (SD), with n = 4.

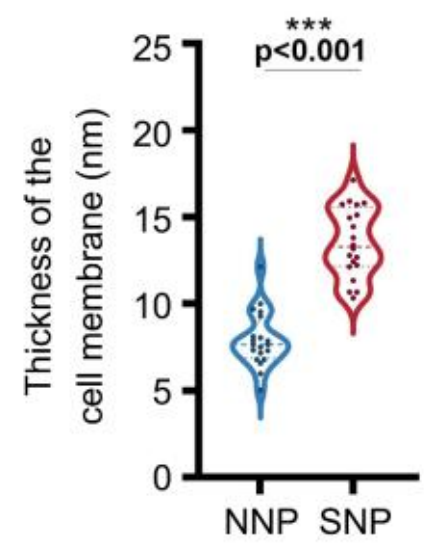

Fig. S7. The thickness of the membrane layer of NNP and SNP. Data are presented as mean ± standard deviation (SD), with n = 20.

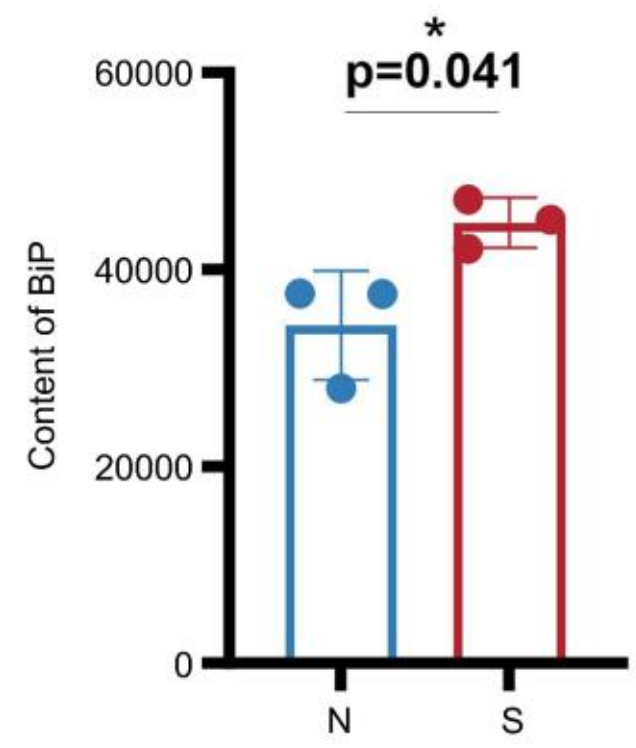

Fig. S8. Content of Bip in normal (N) and senescent (S) 4T1 cells. Data are presented as mean ± standard deviation (SD), with n = 3. Statistical significance ($p<0.05$) was determined by t test.

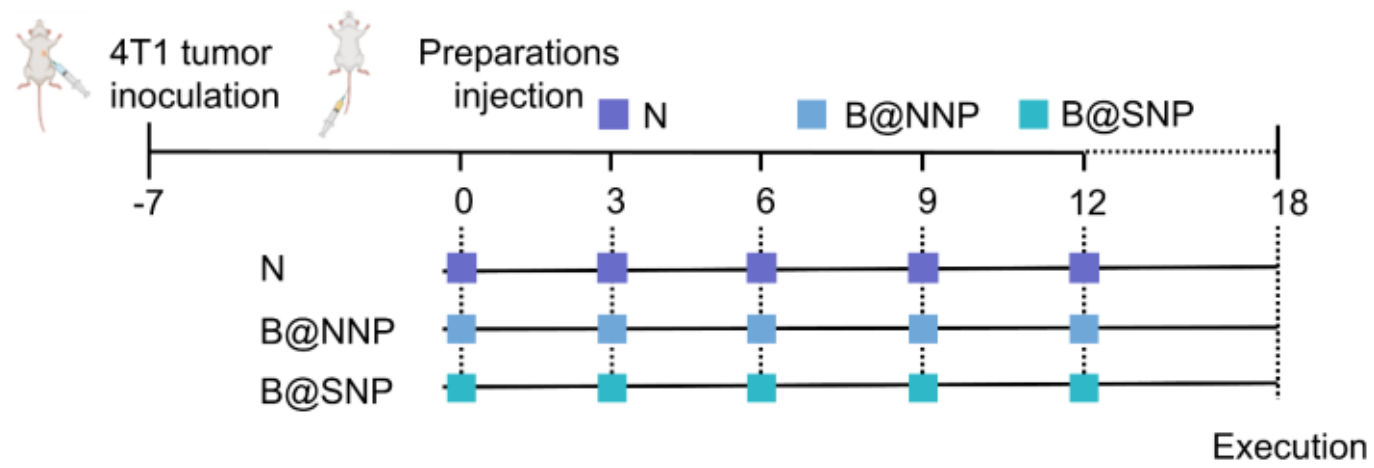

Fig. S9. Therapeutic scheme for the 4T1 metastatic tumor model in BALB/c mice. The illustration was created in BioRender. Tao, J. (2026) https://BioRender.com/o92m76p**.**

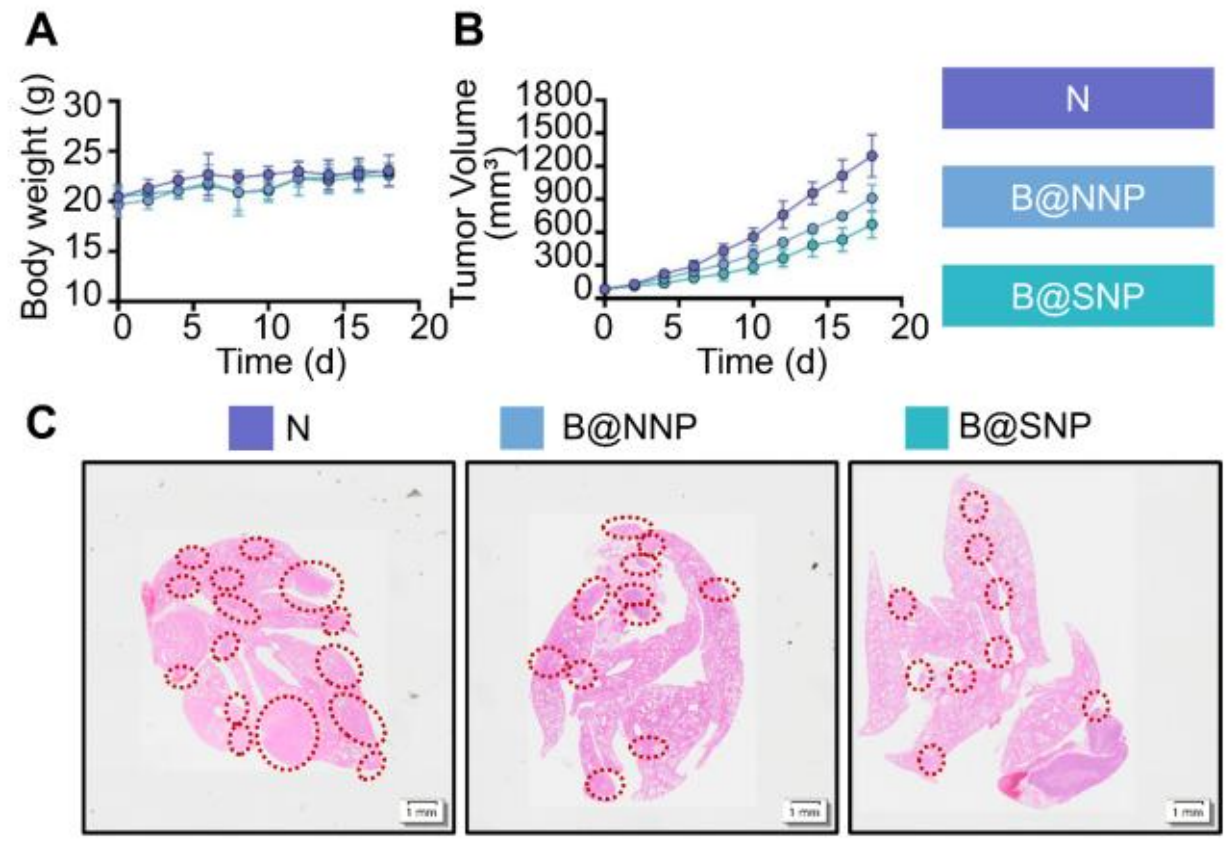

Fig. S10. In vivo anti-metastatic therapeutic efficacy of normal (B@NNP) and senescent (B@SNP) tumor cell membrane-coated nanoparticles. Monitoring of body weight (A) and tumor volume (B) of BALB/c mice within 18 days after administration. (C) Hematoxylin and Eosin (HE) staining images of lung tissues from tumor-bearing mice. Scale bar, 1mm. Data are presented as mean ± standard deviation (SD), with n = 5.

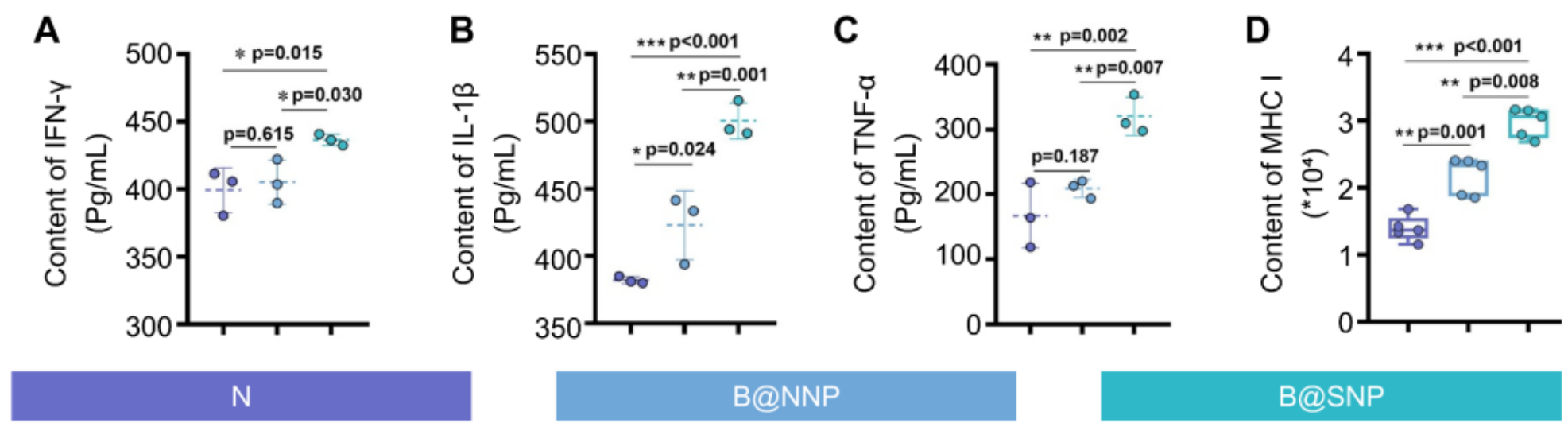

Fig. S11. Cytokine secretion and MHC-I molecule expression in tumor cells after treatment with B@NNP or B@SNP in vivo. (A) IFN-γ, (B) IL-1β, (C) TNF-α, and (D) MHC-I molecules. Data are presented as mean ± standard deviation (SD), with n = 3 or 5. Statistical significance ($p<0.05$) was determined by one-way ANOVA test.

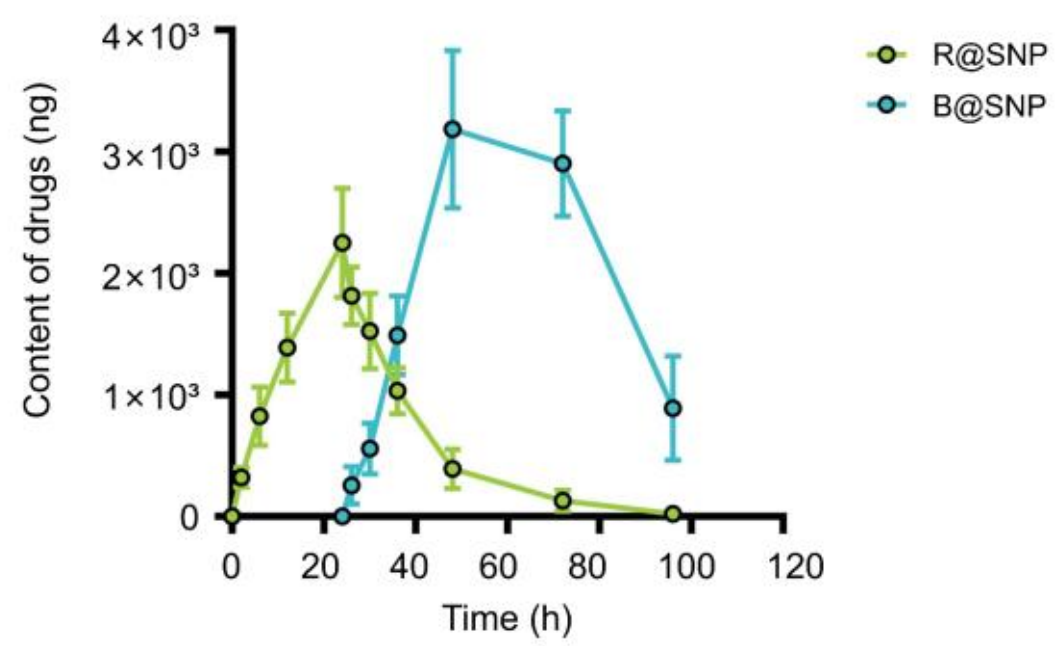

Fig. S12. Tumor distribution profiles of ribociclib and brefeldin A following sequential administration. This figure shows the mass of the drug per gram of tumor tissue. Data are presented as mean ± standard deviation (SD), with n = 4.

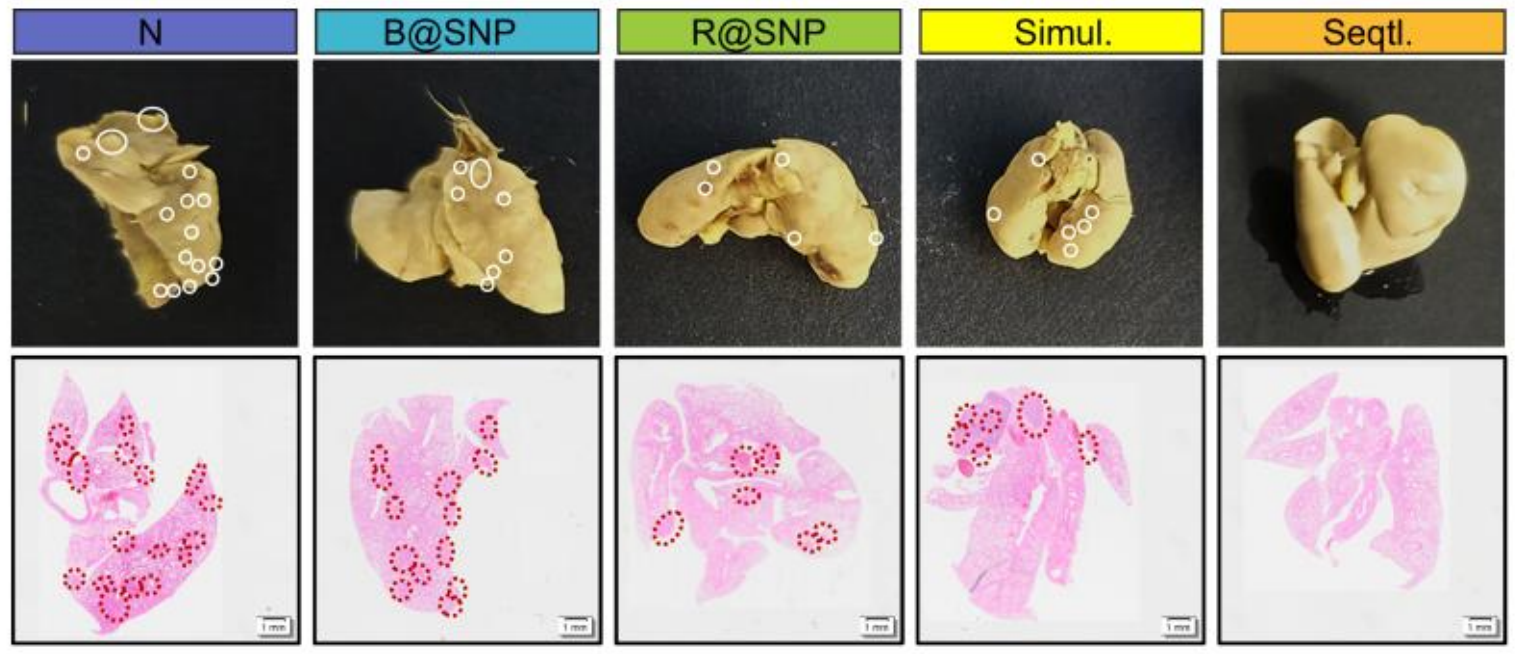

Fig. S13. HE staining images of lung tissues from tumor-bearing mice. Scale bar, 1mm. The lung tissue image shown here is intentionally reused from Fig. 8E to correspond with the associated H&E-stained lung tissue section.

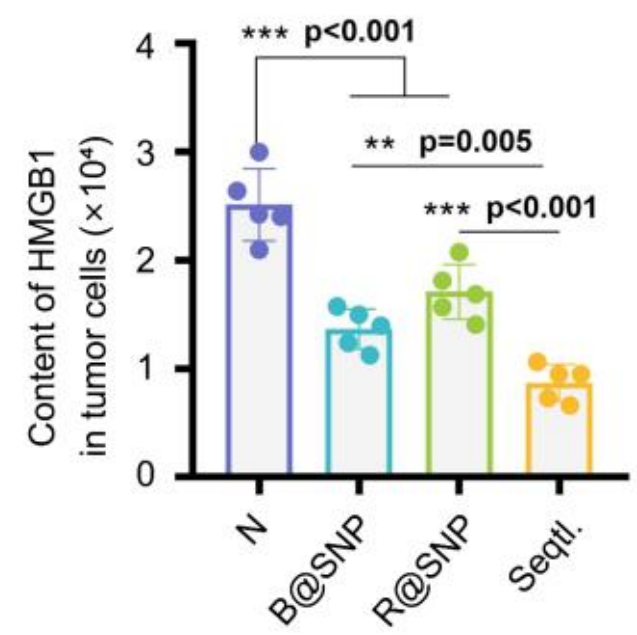

Fig. S14. Content of HMGB1 in tumor cells. Data are presented as mean ± standard deviation (SD), with n = 5. Statistical significance ($p<0.05$) was determined by one-way ANOVA test.

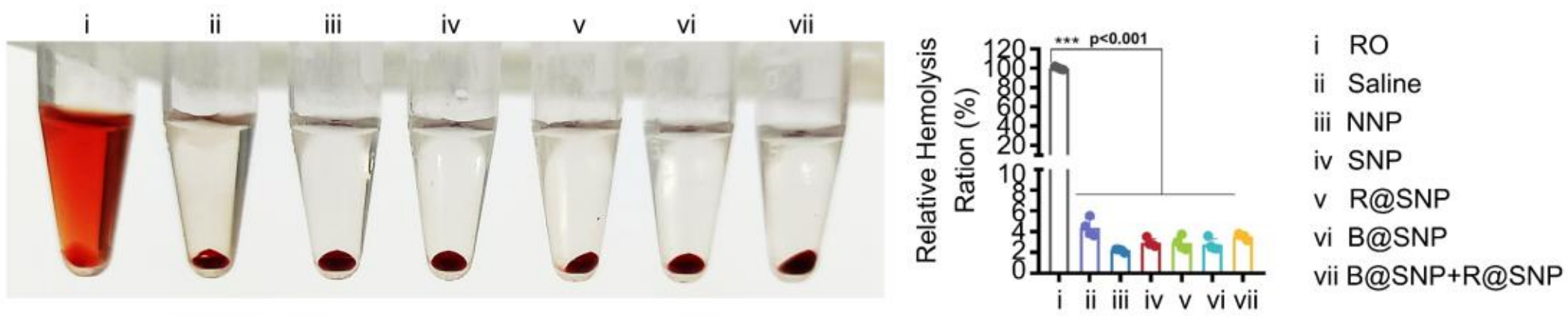

Fig. S15. Hemolysis assay of nanoparticles in all groups. Data are presented as mean ± standard deviation (SD), with n = 3. Statistical significance ($p<0.05$) was determined by one-way ANOVA test.

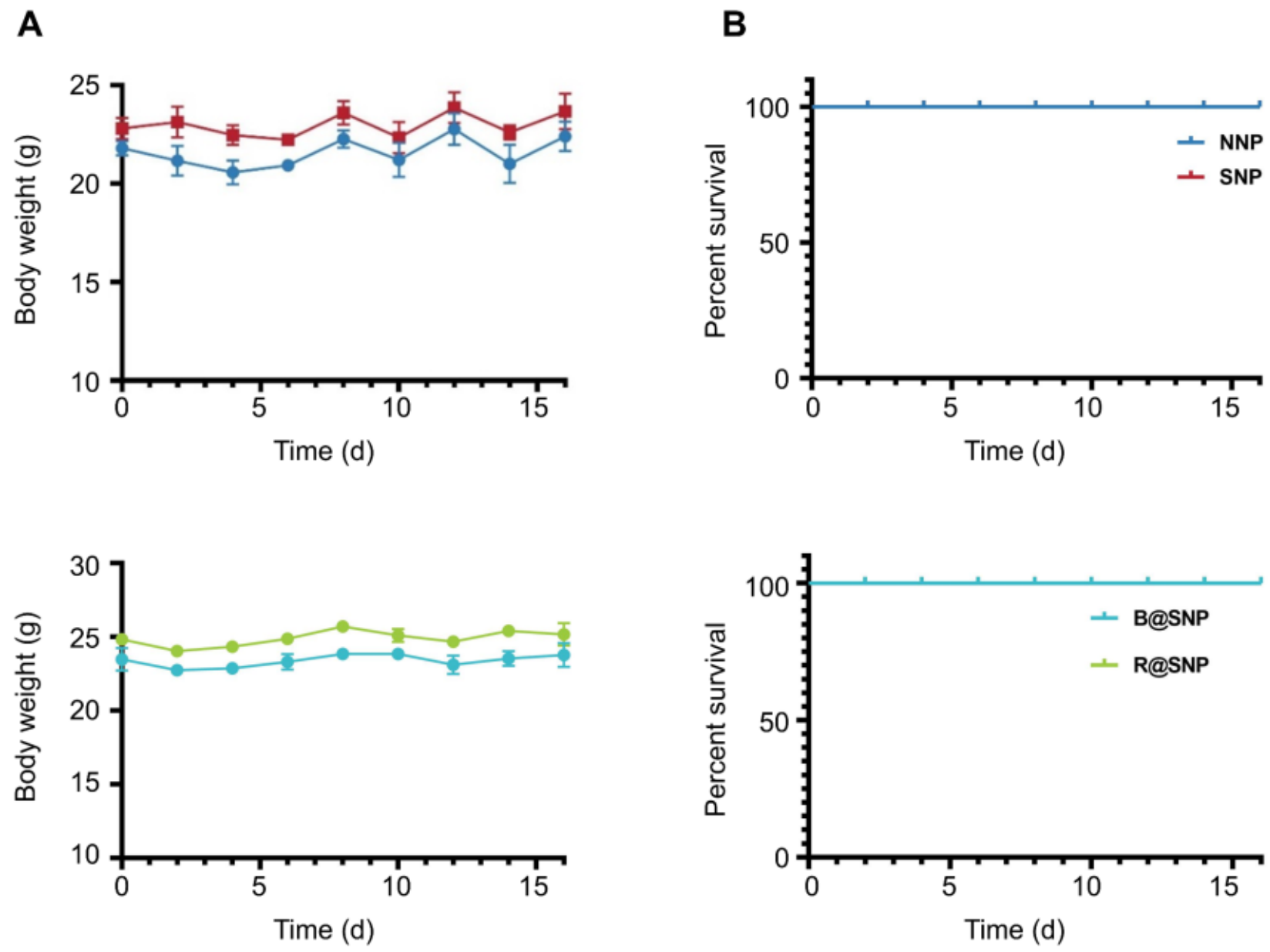

Fig. S16. The toxicity test of nanoparticles in vivo. The administration regimen was consistent with that of the in vivo anti-metastasis efficacy test. (A) Body weight change of mice. (B) Survival curves of mice. Data are presented as mean ± standard deviation (SD), with n = 3.
